# Evolution of multiple sex-chromosomes associated with dynamic genome reshuffling in *Leptidea* wood-white butterflies

**DOI:** 10.1038/s41437-020-0325-9

**Published:** 2020-06-09

**Authors:** Atsuo Yoshido, Jindra Šíchová, Kristýna Pospíšilová, Petr Nguyen, Anna Voleníková, Jan Šafář, Jan Provazník, Roger Vila, František Marec

**Affiliations:** 1 Biology Centre of the Czech Academy of Sciences, Institute of Entomology, Branišovská 31, 370 05 České Budějovice, Czech Republic; 2 Faculty of Science, University of South Bohemia, Branišovská 1760, 370 05 České Budějovice, Czech Republic; 3 Institute of Experimental Botany of the Czech Academy of Sciences, Centre of the Region Hana for Biotechnological and Agricultural Research, Šlechtitelů 31, 779 00 Olomouc, Czech Republic; 4 Institut de Biologia Evolutiva (CSIC-UPF), Pg. Marítim de la Barceloneta 37, 08003 Barcelona, Spain; 5 Present Address: Genomics Core Facility, European Molecular Biology Laboratory, Heidelberg, Germany

**Keywords:** Cytogenetics, Evolutionary biology

## Abstract

Sex-chromosome systems tend to be highly conserved and knowledge about their evolution typically comes from macroevolutionary inference. Rapidly evolving complex sex-chromosome systems represent a rare opportunity to study the mechanisms of sex-chromosome evolution at unprecedented resolution. Three cryptic species of wood-white butterflies—*Leptidea juvernica*, *L. sinapis* and *L. reali*—have each a unique set of multiple sex-chromosomes with 3–4 W and 3–4 Z chromosomes. Using a transcriptome-based microarray for comparative genomic hybridisation (CGH) and a library of bacterial artificial chromosome (BAC) clones, both developed in *L. juvernica*, we identified Z-linked *Leptidea* orthologs of *Bombyx mori* genes and mapped them by fluorescence in situ hybridisation (FISH) with BAC probes on multiple Z chromosomes. In all three species, we determined synteny blocks of autosomal origin and reconstructed the evolution of multiple sex-chromosomes. In addition, we identified W homologues of Z-linked orthologs and characterised their molecular differentiation. Our results suggest that the multiple sex-chromosome system evolved in a common ancestor as a result of dynamic genome reshuffling through repeated rearrangements between the sex chromosomes and autosomes, including translocations, fusions and fissions. Thus, the initial formation of neo-sex chromosomes could not have played a role in reproductive isolation between these *Leptidea* species. However, the subsequent species-specific fissions of several neo-sex chromosomes could have contributed to their reproductive isolation. Then, significantly increased numbers of Z-linked genes and independent neo-W chromosome degeneration could accelerate the accumulation of genetic incompatibilities between populations and promote their divergence resulting in speciation.

## Introduction

Sex chromosomes (XY/XX in male heterogamety and WZ/ZZ in female heterogamety) are known to play an important role in fundamental evolutionary processes, such as sex determination, inheritance of sex-specific traits, adaptation and speciation. A large contribution of the X chromosome to reproductive isolation (the so-called ‘large X-effect’) is well established, especially in *Drosophila* species (Presgraves 2008). In some organisms with female heterogamety, Z-linked genes or traits also contribute significantly to speciation and adaptation, referred to as the ‘large Z-effect’ (Qvarnström and Bailey 2009). Sex-limited Y or W chromosomes are frequently associated with sex-specific traits. For example, in some organisms, these chromosomes carry primary sex-determining signals (Bachtrog et al. 2014). In addition, several studies suggest that the Y chromosome plays a role in reproductive isolation of some organisms (Sweigart 2010; Campbell et al. 2012). The chicken W chromosome affects female fertility traits as well (Moghadam et al. 2012). Due to these properties, turnover of sex chromosomes by rearrangements with autosomes may facilitate adaptation and promote speciation (Kitano et al. 2009; Kitano and Peichel 2012; Nguyen et al. 2013; Graves 2016). Moreover, the sex chromosomes themselves are the source of intralocus and intragenomic conflicts that may even cause the turnover of sex chromosomes (Mank et al. 2014). Neo-sex chromosomes and multiple sex-chromosomes, originating from fusions and/or translocations between sex chromosomes and autosomes, occur in species with both XY and WZ systems (Marec et al. 2010; Pennell et al. 2015). Exceptionally, repeated sex chromosome-autosome translocations can generate even more complex multiple sex-chromosomes like X_1–5_Y_1–5_/X_1–5_X_1–5_ in platypus (Rens et al. 2004). Thus, the evolution and molecular differentiation of sex chromosomes are among the most intriguing questions of evolutionary genetics.

Accumulating evidence suggests that sex chromosomes of moths and butterflies (Lepidoptera), the largest group of animals with holokinetic chromosomes and female heterogamety, also play a disproportionate role in speciation compared with autosomes. In a number of lepidopteran species, Z-linked genes significantly contribute to the formation of prezygotic and/or postzygotic reproductive barriers between different strains and closely related species (Sperling 1994; Naisbit et al. 2002; Presgraves 2002; Dopman et al. 2005; Kost et al. 2016). In addition, recent results suggest that a sex-linked inversion promotes divergence and facilitates speciation by suppressing recombination between Z chromosomes of two strains of the European corn borer moth, *Ostrinia nubilalis* (Wadsworth et al. 2015). Besides the common WZ/ZZ (♀/♂) sex chromosomes, WZ_1_Z_2_ and W_1_W_2_Z multiple sex-chromosome systems were found only in several distantly related lepidopteran taxa (Traut et al. 2007; Marec et al. 2010; Sahara et al. 2012). However, neo-sex chromosomes formed by fusion of both W and Z chromosomes with autosomes are much more common in Lepidoptera than previously thought (Nguyen et al. 2013; Mongue et al. 2017; Carabajal Paladino et al. 2019). Transfer of the whole set of autosomal genes under Z-linkage, as in multiple or neo-sex chromosomes, can greatly contribute to ecological specialisation, reproductive isolation and speciation in Lepidoptera (Yoshido et al. 2011a; Nguyen et al. 2013; Carabajal Paladino et al. 2019). This is demonstrated by the neo-W chromosome of the African queen butterfly, *Danaus chrysippus*, which drives speciation across the hybrid zone by linking the colour pattern and male killing caused by an endosymbiotic bacterium, *Spiroplasma ixodeti* (Smith et al. 2016; Traut et al. 2017).

Wood-white butterflies of the genus *Leptidea* (Pieridae) constitute an excellent model for studying the role of chromosome rearrangements in speciation. Especially three species with mainly Western Palaearctic distribution—*L. juvernica*, *L. sinapis* and *L. reali*—represent one of the most striking examples of cryptic diversity in Eurasian butterflies (Dincă et al. 2011). This triplet of species has evolved strong pre-mating reproductive barriers in their sympatric and allopatric populations (Friberg et al. 2008; Dincă et al. 2013). In addition, chromosome numbers vary greatly between and even within the species due to multiple chromosome fusions and fissions (Dincă et al. 2011; Lukhtanov et al. 2011, 2018; Šíchová et al. 2015). Genome sequencing showed that the genome assembly of *L. sinapis* (643 Mb) is one of the largest in Lepidoptera studied so far, and variations in genome size between and within *Leptidea* species have been documented (Talla et al. 2017).

Previous studies showed that each of the four *Leptidea* species examined have a unique, species-specific and complex system of multiple sex-chromosomes: W_1–3_Z_1–4_/Z_1–4_Z_1–4_ in *L. juvernica* (♀/♂), W_1–3_Z_1–3_/Z_1–3_Z_1–3_ in *L. sinapis*, W_1–4_Z_1–4_/Z_1–4_Z_1–4_ in *L. reali* and W_1–3_Z_1–6_ /Z_1–6_Z_1–6_ in *L. amurensis* (Šíchová et al. 2015, 2016). Despite fluctuating chromosome numbers, even between siblings of individual species, the sex-chromosome systems seem stable in each species. Thus, these closely related species provide a unique opportunity to address the role of sex-chromosome rearrangements in the formation of reproductive barriers between their populations. However, little is known about the composition and origin of these multiple sex-chromosomes, because in such complex systems it is difficult to identify all sex chromosomes simply from genome assembly.

In this study, we cytogenetically identified Z chromosomes in three cryptic *Leptidea* species (*L. juvernica*, *L. sinapis* and *L. reali*) and reconstructed the evolution of their multiple sex-chromosome systems by comparative mapping of sex-linked genes. For this purpose we have developed a couple of genomic tools in *L. juvernica*, namely a female transcriptome-based microarray for comparative genomic hybridisation (CGH) and a bacterial artificial chromosome (BAC) library from females. BAC probes containing *Leptidea* orthologs of *Bombyx mori* genes identified all Z chromosomes in three *Leptidea* species. Furthermore, we analysed several BAC clones derived from the W chromosomes of *L. juvernica*, which allowed us to identify female-specific sequences and W-linked genes of autosomal origin.

## Materials and methods

### Specimens

Adult specimens of *Leptidea juvernica* and *L. sinapis* were collected in several localities in the Czech Republic whereas *L. reali* was collected in the Montseny area near Barcelona, Spain. The taxonomic determination of specimens used was verified by morphometric analysis of their genitalia and sequencing of a mitochondrial gene, cytochrome c oxidase subunit 1, as described in Šíchová et al. (2015). Fertilised females were individually kept in plastic containers to lay eggs. Hatched larvae of all three species were reared on one of their host plants, *Lotus corniculatus*, at room temperature and a natural day/night regime.

### RNA sequencing and female transcriptome assembly in *Leptidea juvernica*

Total RNA was extracted from a homogenised female larva of *L. juvernica* (gut removed) using an RNA Blue reagent (Top-Bio, Prague, Czech Republic) according to the manufacturer’s protocol. An mRNA-seq library was constructed and sequenced using the Illumina HiSeq2000 platform by EMBL Genomics Core Facility (Heidelberg, Germany). Generated raw 100-bp paired-end reads were checked by FastQC (Andrews 2010) and trimmed and quality filtered by Trimmomatic version 0.30 (‘LEADING:5 TRAILING:5 SLIDINGWINDOW:4:20’; Bolger et al. 2014). Transcriptome sequence was then assembled de novo by SOAPdenovo-trans-127mer with multiple odd k-mer sizes ranging from 21 to 81 (Xie et al. 2014) and Trinity (Haas et al. 2013). The resulting assemblies were merged and redundancy was removed by the EvidentialGene pipeline (Gilbert 2013).

### Array-CGH analysis in *Leptidea juvernica*

To identify sex-linked genes in *L. juvernica*, microarray-based comparative genomic hybridisation (array-CGH) was performed according to the method described in Baker and Wilkinson (2010) (for details, see Supplementary Methods). Briefly, we searched for *Leptidea* orthologs of *Bombyx mori* genes in the EvidentialGene output using HaMStR (Ebersberger et al. 2009). The *Leptidea* orthologs were used to design 60-mer oligonucleotide probes for custom-made microarray slides using Agilent Technologies eArray design wizard. DNA digestion, labelling and array-CGH were performed by GenLabs (Prague, Czech Republic) following a protocol for Agilent oligonucleotide array-based CGH for genomic DNA analysis.

### Construction of BAC library in *Leptidea juvernica* females

A BAC library was constructed from female pupae of *L. juvernica*. The procedure followed the modified protocol described previously (Šafář et al. 2010). Briefly, high molecular weight DNA isolated from female pupae and embedded in agarose plugs was partially digested with restriction enzyme *Hin*dIII. The digested DNA was separated by pulsed field gel electrophoresis (two size-selection steps); 100–300-kb fragments were then isolated from the gel and ligated into a dephosphorylated vector pAGIBAC5. Recombinant BACs were transformed into *E. coli* strain MegaX DH10B T1 (Invitrogen, Carlsbad, CA, USA) by electroporation. In total, 36,864 clones were picked by an automatic robotic station Q-BOT, ordered in 384-well microtiter plates, and stored at −80 °C. The average insert size in BAC clones was a~125 kb. A master copy of the BAC library, named LjufhA, is stored at the Centre of Plant Structural and Functional Genomics in Olomouc, Czech Republic. The master copy was used for preparation of two working copies. Subsequently, clones from a working copy of the LjufhA BAC library were used to prepare 3 DNA pools. Briefly, a liquid handling system Biomek NX (Beckman Coulter, Brea, CA, USA) was used for pooling 384 clones of each plate (‘plate pool’), for pooling rows from 384-well plates (‘row pool’), and for pooling columns from 384-well plates (‘column pool’). DNA from pooled clones was isolated by standard alkaline lysis and ordered in 96-well microtiter plates for further PCR screening.

### BAC screening for FISH mapping

Sequence information of *Leptidea* orthologs of *B. mori* genes was obtained from the transcriptome assembly of *L. juvernica* females. By blast searching gene models in KAIKObase (http://sgp.dna.affrc.go.jp/KAIKObase/) against the *L. juvernica* transcriptome, selected *Leptidea* orthologs of *B. mori* genes were identified. We then designed primer sets to identify BAC clones containing the respective *Leptidea* orthologs from the BAC library (Supplementary Table S1). For BAC screening, we used a 3-step PCR-based screening method as described previously (Yasukochi 2002; Yoshido et al. 2015). Each reaction mixture was composed of 1.0 μl of template BAC-DNA pools, 10 pmol of each primer, 0.25 U of One*Taq* DNA Polymerase and 2.0 μl of 5× One*Taq* Standard Reaction Buffer (New England BioLabs, Ipswich, MA, USA). PCR amplifications were conducted under the following conditions: initial denaturation for 5 min at 94 °C, 40 cycles of denaturation for 30 s at 94 °C, annealing for 30 s at 55–60 °C, elongation for 30–180 s at 68 °C and final elongation for 1 min at 72 °C. PCR products were loaded on 1.0–2.0% agarose gel in TAE buffer. Gels were stained with ethidium bromide and photographed under UV light.

### BAC-FISH mapping

All required procedures are described in detail in Supplementary Methods. Spread preparations of meiotic chromosomes were obtained from gonads (ovaries and testes) and mitotic chromosomes from wing imaginal discs of the last instar larvae as described in Šíchová et al. (2015) and Yoshido et al. (2015). For mapping of gene orthologs on a particular chromosome, several rounds of two-colour BAC-FISH were carried out following the procedure and reprobing protocol described in Yoshido et al. (2015). Extracted BAC-DNAs were labelled by nick translation using a mixture of DNase I and DNA polymerase I with either aminoallyl-dUTP-Cy3 or fluorescein-12-dUTP and BAC probes hybridised to chromosomes using the BAC-FISH mapping protocol (Yoshido et al. 2015).

### Analysis of BAC clones derived from W chromosomes and W homologues of Z-linked orthologs

To find BAC clones derived from W chromosomes in *L. juvernica*, we screened the BAC library using FISH-based screening (Supplementary Fig. S1). Extracted BAC-DNAs of two clones derived from W chromosomes of *L. juvernica* (see ‘Results’) were digested with restriction enzyme *Hin*dIII, and several digested DNA fragments (700–5000 bp) were sub-cloned into pJET1.2/blunt Cloning Vector using the CloneJET PCR Cloning Kit (Thermo Fisher Scientific, Waltham, MA, USA). The plasmids obtained were transformed into *Escherichia coli* DH5α strain. Plasmid DNAs were extracted using the NucleoSpin Plasmid kit (Macherey-Nagel, Düren, Germany) and sequenced using universal primers. Analysis of W homologues of Z-linked orthologs of the *Uch5l* and *Gst8* genes was performed by PCR and sequencing as described in Supplementary Methods.

### Quantitative real-time PCR (qPCR)

To compare a relative gene dose of *Leptidea* orthologs between females and males, we performed quantitative real-time PCR (qPCR) using gDNAs as templates according to the method described in Nguyen et al. (2013) with slight modifications. For qPCR, gDNAs were extracted separately from three females and three males of *L. juvernica*, *L. sinapis*, and *L. reali* by standard phenol–chloroform procedure. Target and reference genes were analysed simultaneously in three different specimens for each sex. The *Leptidea* ortholog of the *B. mori RpS5* gene was used as an autosomal reference gene. Primer sets for respective target and reference genes are listed in Supplementary Table S1 (asterisk) or Table S5. The qPCR was performed using a reaction mixture composed of 0.5–20 ng of template gDNA, 0.5 pmol of each primer and 5 μl of Xceed qPCR SG 2× Mix Lo-Rox (Institute of Applied Biotechnologies, Prague, Czech Republic). To calculate the amplification efficiency (*E*), 4 points of 4 times dilution series were used. The obtained data were processed using CFX Manager Software (Bio-Rad). The PCR reaction was carried out using the C1000 Thermal cycler CFX96 Real-Time System (Bio-Rad, Hercules, CA, USA). The female-to-male relative dose ratio of the target gene was determined by comparison with the autosomal reference gene using a formula of Pfaffl (2001) and statistically analysed by unpaired two-tailed *t* test for unequal variances.

## Results

### Array-CGH in *Leptidea juvernica*

**Fig. 1:**
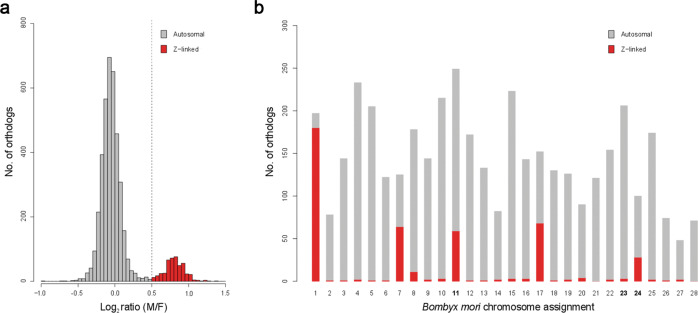
Array-CGH in *Leptidea juvernica*. **a** Distribution of CGH log_2_ ratio of male-to-female signal intensities. The peak centred at −0.1 (grey) corresponds to putative autosomal orthologs while bins with values >0.5 (red) forming the smaller peak comprise putative Z-linked orthologs. A total of 4252 orthologs are presented. **b** Autosomal (grey) and sex-linked (red) *L. juvernica* orthologs and their chromosome assignment in the *Bombyx mori* reference genome; *B. mori* chromosomes of fusion origin are in bold.

To identify candidate orthologs located on *L. juvernica* Z chromosomes, we carried out array-CGH (Fig. 1). After filtering, *L. juvernica* log_2_ ratio values of male-to-female signal intensities were obtained for 4252 orthologs. The values averaged across four replicas clearly showed a bimodal distribution with an autosomal peak centred at −0.1 (Fig. 1a). Using a cut-off value of 0.5, we identified 454 putative Z-linked orthologs. Chromosomal location of their *B. mori* counterparts revealed that the identified *L. juvernica* Z-linked orthologs contain vast majority of genes assigned to *B. mori* Z-chromosome (chr. 1) and are further enriched on *B. mori* chr. 7, chr. 8, chr. 11, chr. 17 and chr. 24 (Fig. 1b).

### BAC-FISH mapping of Z_1_ chromosome

**Fig. 2:**
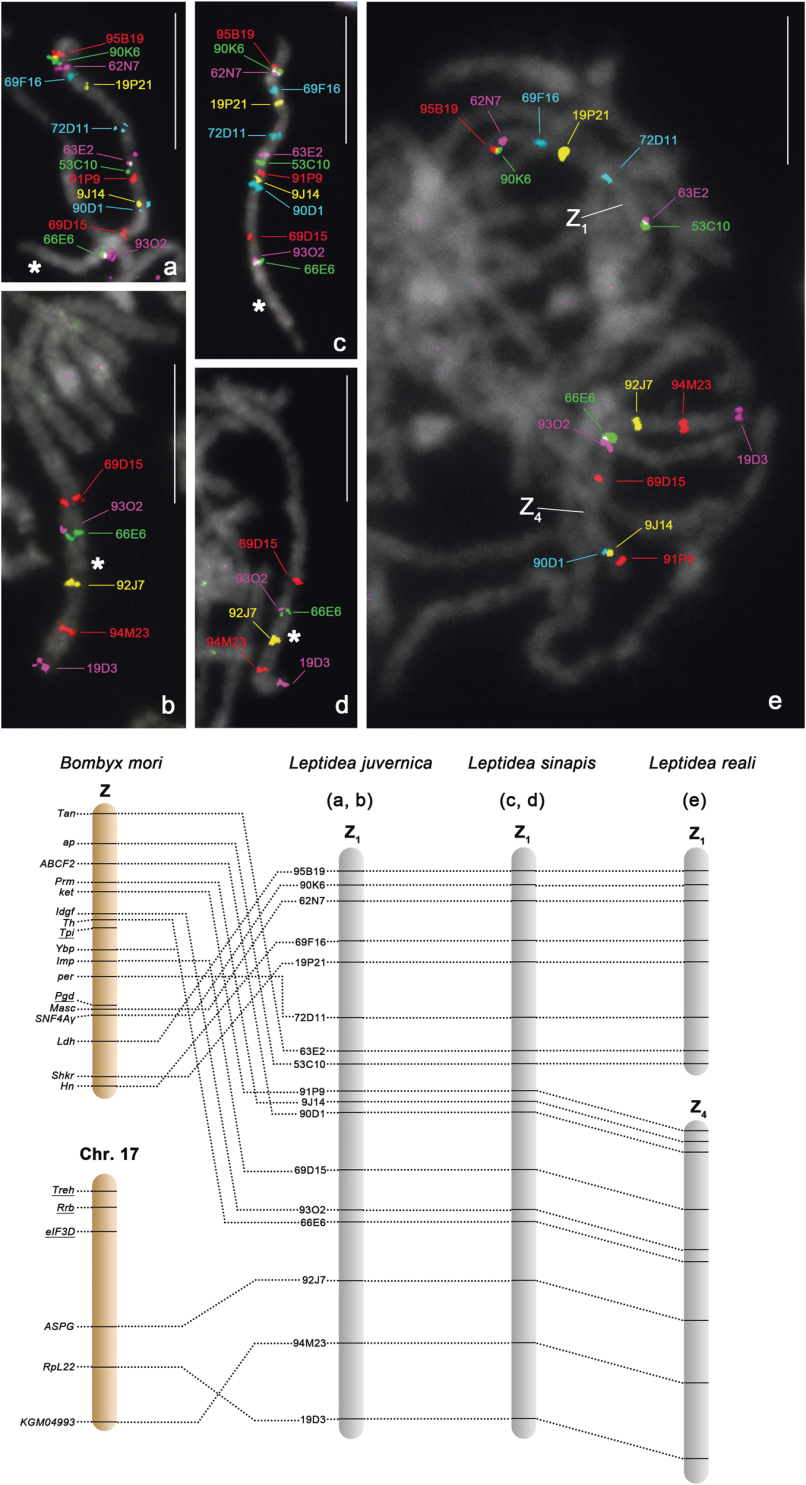
BAC-FISH mapping of Z_1_ chromosome in *Leptidea juvernica* and *L. sinapis* and Z_1_ and Z_4_ chromosomes in *L. reali*. Upper panel: male pachytene chromosomes showing hybridisation signals of Cy3- or fluorescein-labelled 14 BAC probes containing *Leptidea* orthologs of *Bombyx mori* Z-linked and 3 BAC probes of chromosome 17-linked genes. Chromosomes were stained with DAPI (grey). Bar = 10 μm. Asterisk indicates the beginning of region orthologous to *B. mori* chr. 17. (**a**, **b**) *L. juvernica*. (**c**, **d**) *L. sinapis*. (**e**) *L. reali*. Lower panel: schematic illustrations of comparative gene mapping between three *Leptidea* species and *B. mori*. The underlined genes on *B. mori* chromosomes indicate orthologs that mapped to autosomes in *Leptidea* species (Supplementary Fig. S3a).

We identified 19 BAC clones containing *Leptidea* orthologs of 17 *B. mori* Z-linked genes (Supplementary Table S1) and mapped them to *L. juvernica* chromosomes. Fourteen out of nineteen prepared BAC probes mapped to a single bivalent in *L. juvernica* males (Fig. 2a and Supplementary Table S1). This bivalent is evidently a Z-chromosome pair, since two representative probes out of these 14, BAC 62N7 and 91P9, containing orthologs of *Masc* and *ket* genes, respectively, mapped both to the same element of the sex-chromosome multivalent in *L. juvernica* females (Fig. 5a). We named this element as Z_1_ chromosome. One of the probes, BAC 9J14 containing the *Leptidea* ortholog of *B. mori Prm*, hybridised not only to Z_1_ but also to one autosomal bivalent, suggesting duplication of the Z_1_ region carrying this gene (Supplementary Fig. S2a). The remaining four probes, BAC 91C3 containing the *Pgd* ortholog, 96D19, 66L7 and 62O17 containing each different size-fragment of the *Tpi* ortholog (Supplementary Table S1), mapped to autosomal bivalents in *L. juvernica* females (Supplementary Text 1 and Supplementary Fig. S2b, c). These results suggest that the orthologs of these two *B. mori* Z-linked genes, *Pgd* and *Tpi*, are not Z-linked in *L. juvernica* and the *Tpi* ortholog is not a single copy gene in the genome. Gene movement out of the *L. juvernica* Z_1_ chromosome, such as *Pgd* translocation and *Tpi* translocation followed by duplication, is better documented by results of array-CGH, which showed that only 17 of the 197 tested orthologs (i.e., 8.6%) of *B. mori* Z-linked genes are autosomal (Fig. 1b). Regarding *Pgd*, the autosomal location of this ortholog is fully consistent with a −0.0125 CGH log_2_ ratio of the male-to-female signal intensities. The orthologs of *Prm* and *Tpi* were not included in the microarray.

FISH mapping of 14 BAC probes containing *Leptidea* orthologs of *B. mori* Z-linked genes showed that the probes did not cover the whole length of the *L. juvernica* Z_1_ chromosome (Fig. 2a, asterisk). Thus, we tested BAC clones containing putative sex-linked *Leptidea* orthologs of *B. mori* autosomal genes identified by array-CGH (Fig. 1b). Three BAC probes, 19D3, 94M23 and 92J7, containing orthologs of *B. mori* chr. 17 genes, also mapped to Z_1_ in *L. juvernica* males (Fig. 2b). However, three BAC probes, 66E20, 65E15 and 93F8, containing putative autosomal orthologs of *B. mori* chr. 17 genes, mapped to two autosomes in *L. juvernica* males (Fig. 2, underlined genes and Supplementary Fig. S3a). These results suggest that the Z_1_ chromosome of *L. juvernica* consists of *Leptidea* orthologs of most of the *B. mori* Z-linked genes and part of the *B. mori* chr. 17 genes.

To verify the conservation of the Z_1_ chromosome in *Leptidea*, Z_1_-derived BAC probes were cross-hybridised to chromosomes of two other species, *L. sinapis* and *L. reali*. In *L. sinapis*, 17 Z_1_-derived BAC probes mapped to a single bivalent in males and even in the same order as in the Z_1_ chromosome of *L. juvernica* (Fig. 2c, d). Three representative BAC probes out of these 17, 62N7, 91P9 and 92J7, mapped to the sex-chromosome multivalent in *L. sinapis* females (Fig. 5c), thus confirming that the BAC-FISH-identified bivalent in *L. sinapis* males is a pair of Z_1_ chromosomes that are orthologous to the Z_1_ chromosome in *L. juvernica*. In contrast, 8 of the 17 Z_1_-derived BAC probes of *L. juvernica* mapped to one bivalent and 9 probes to another bivalent in *L. reali* males (Fig. 2e). This result showed that in *L. reali*, the Z_1_ of *L. juvernica* and *L. sinapis* split into two chromosomes in the region between the *ap* and *ket* orthologs (BAC probes 53C10 and 91P9, respectively) (Fig. 2). The order of 17 BAC loci in the respective bivalents was conserved in all three species. One of the BAC probes, 62N7, representing one of the two mapped bivalents and two BAC probes, 91P9 and 92J7, representing the other bivalent hybridised to the sex-chromosome multivalent in *L. reali* females (Fig. 5e). This result clearly showed that the mapped bivalents represent two of the four Z chromosomes in *L. reali*. We marked them as Z_1_ and Z_4_ chromosomes (Fig. 2).

### BAC-FISH mapping of Z_2_ and Z_3_ chromosomes

**Fig. 3:**
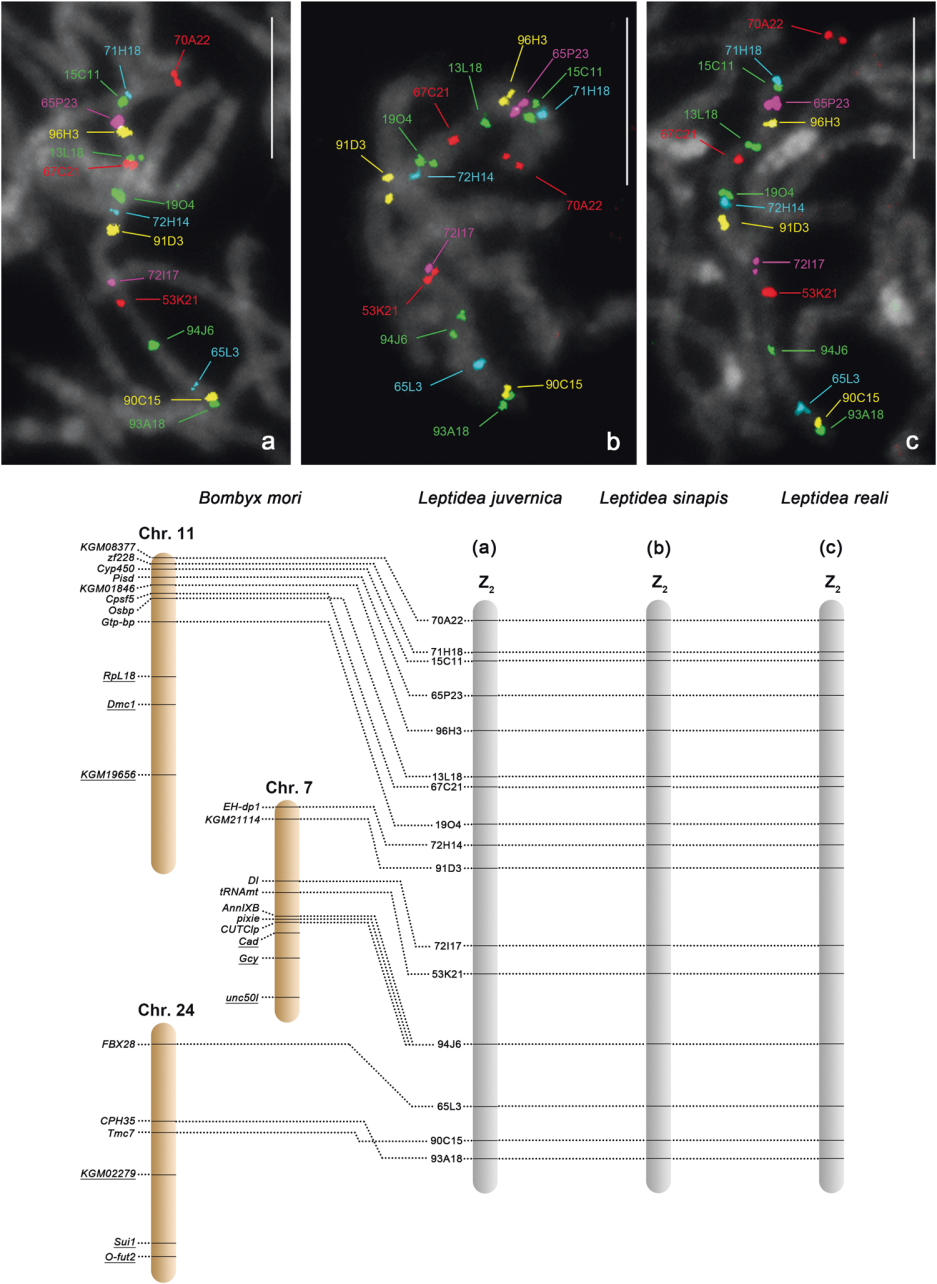
BAC-FISH mapping of Z_2_ chromosome in three *Leptidea* species. Upper panel: male pachytene chromosomes showing hybridisation signals of Cy3- or fluorescein-labelled 16 BAC probes containing *Leptidea* orthologs of *Bombyx mori* chr. 7, chr. 11 and chr. 24 genes. Chromosomes were stained with DAPI (grey). Bar = 10 μm. **a** *L. juvernica*. **b** *L. sinapis*. **c** *L. reali*. Lower panel: schematic illustrations of comparative gene mapping between three *Leptidea* species and *B. mori*. The underlined genes on *B. mori* chromosomes indicate orthologs that mapped to autosomes in *Leptidea* species (Supplementary Fig. S3b, c).

To determine the origin of the other two Z chromosomes in *L. juvernica*, we performed FISH mapping of BAC clones containing putative sex-linked *Leptidea* orthologs of *B. mori* autosomal genes that were identified by array-CGH (Fig. 1b). Sixteen BAC probes containing orthologs of *B. mori* chr. 7, chr. 11 and chr. 24 genes mapped to a single bivalent in *L. juvernica* males and covered the entire length of the bivalent (Fig. 3a). One representative BAC probe (94J6) of these 16 hybridised to one chromosome in the sex-chromosome multivalent in *L. juvernica* females (Fig. 5a). Thus, the male bivalent identified by BAC-FISH corresponds to another pair of four Z chromosomes in *L. juvernica*, referred to as the Z_2_ chromosome. Another BAC-FISH experiment showed that three respective BAC probes containing autosomal orthologs of *B. mori* chr. 7, chr. 11 and chr. 24 genes indeed map to autosomes in *L. juvernica* males (see the underlined genes in Fig. 3 and Supplementary Fig. S3b, c). Based on the above results, we concluded that the Z_2_ chromosome of *L. juvernica* consists of three segments corresponding to parts of *B. mori* chr. 11, chr. 7 and chr. 24 (Fig. 3, lower panel).

All 16 BAC probes that mapped to the Z_2_ chromosome of *L. juvernica* also mapped to the respective bivalent in *L. sinapis* and *L. reali* males. In addition, the order of mapped probes was also fully conserved in all three species (Fig. 3b, c). The representative BAC 94J6 probe of these 16 also hybridised to one chromosome of the sex-chromosome multivalent in *L. sinapis* and *L. reali* females (Fig. 5c, e), confirming that it is indeed a Z_2_ chromosome in both species.

**Fig. 4:**
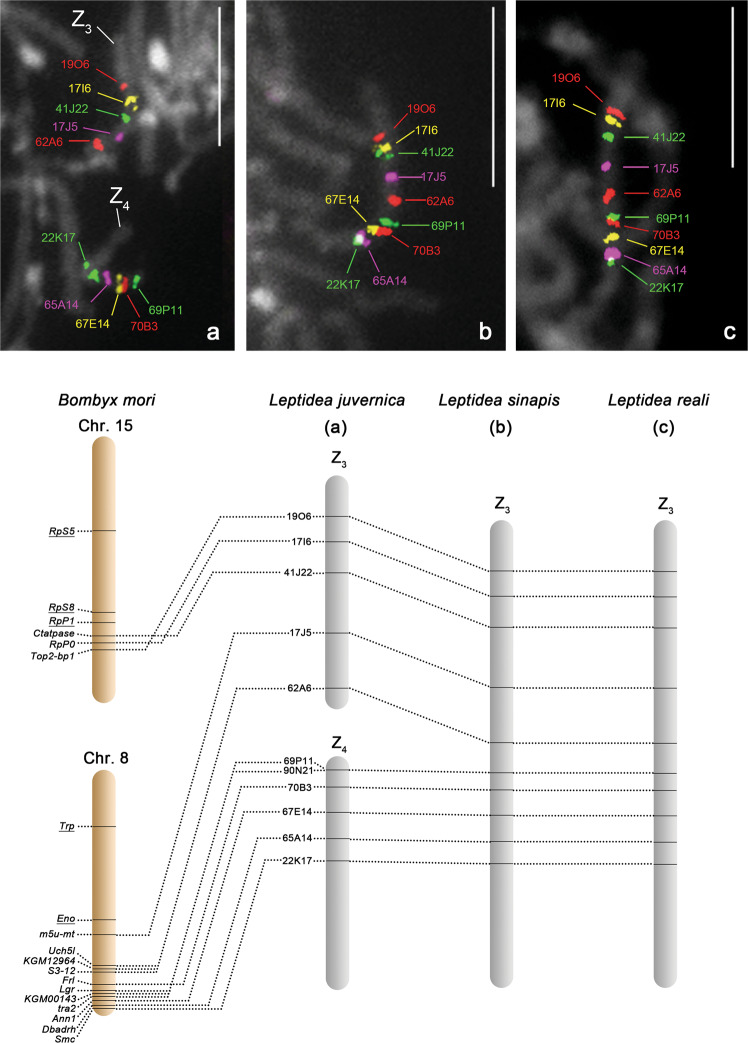
BAC-FISH mapping of Z_3_ and Z_4_ chromosomes in *Leptidea juvernica* and Z_3_ chromosome in *L. sinapis* and *L. reali*. Upper panel: male pachytene chromosomes showing hybridisation signals of Cy3- or fluorescein-labelled ten BAC probes containing *Leptidea* orthologs of *Bombyx mori* chr. 8 and chr. 15 genes. Chromosomes were stained with DAPI (grey). Bar = 10 μm. **a** *L. juvernica*. **b** *L. sinapis*. **c** *L. reali*. Lower panel: schematic illustrations of comparative gene mapping between three *Leptidea* species and *B. mori*. The underlined genes on *B. mori* chromosomes indicate orthologs that mapped to autosomes in *Leptidea* species (Supplementary Fig. S3c).

**Fig. 5:**
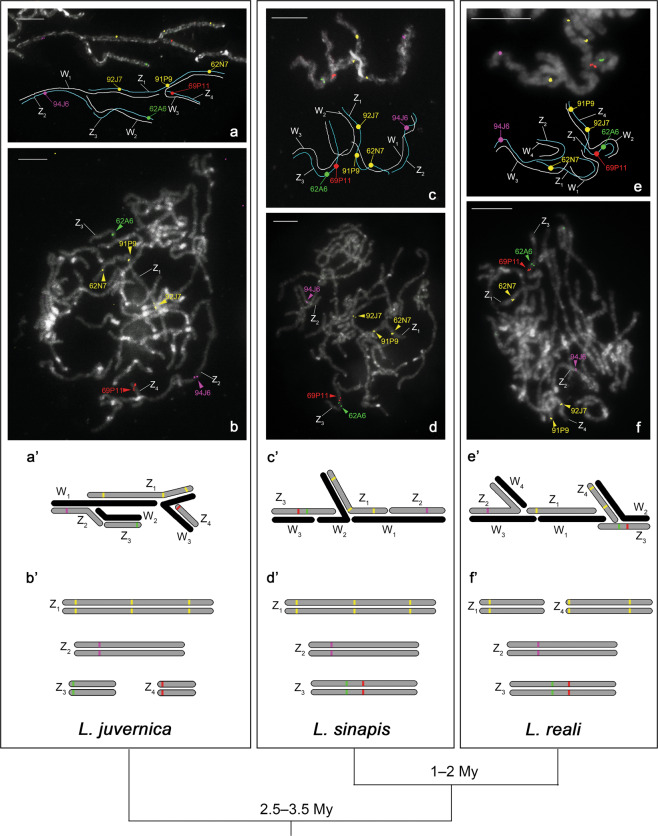
BAC-FISH analyses of multiple sex-chromosomes in three *Leptidea* species. Upper panel (**a**–**f**): FISH mapping of BAC clones representing respective Z chromosomes in female and male pachytene chromosomes. BAC clones (see Supplementary Table S1) mapping to different Z chromosomes are marked with a different colour. Chromosomes were stained with DAPI (grey). Bar = 10 μm. (**a** BAC-FISH image and schematic drawing of the sex-chromosome multivalent in female pachytene of *L. juvernica*. **b** Male pachytene complement of *L. juvernica*. **c** BAC-FISH image and schematic drawing of the sex-chromosome multivalent in female pachytene of *L. sinapis*. **d** Male pachytene complement of *L. sinapis*. **e** BAC-FISH image and schematic drawing of the sex-chromosome multivalent in female pachytene of *L. reali*. **f** Male pachytene complement of *L. reali*. Lower panel (**a’**–**f’**): schematic illustrations of multiple sex-chromosomes in three *Leptidea* species based on BAC-FISH results shown in upper panel. **a’** *L. juvernica* female. **b’** *L. juvernica* male. **c’** *L. sinapis* female. **d’** *L. sinapis* male. **e’** *L. reali* female. **f’** *L. reali* male. Z and W chromosomes are coloured grey and black, respectively. The phylogenetic relationships and estimated divergence times (My, million years) between the species are shown below the lower panel (Šíchová et al. 2015; Talla et al. 2017, 2019a).

Eight BAC probes containing orthologs of *B. mori* chr. 8 genes mapped to two bivalents in *L. juvernica* males (Fig. 4a). One of the BAC probes (62A6), representing one of the two bivalents and another BAC probe (69P11), representing the other bivalent hybridised to the sex-chromosome multivalent in *L. juvernica* females (Fig. 5a), suggesting that both bivalents correspond to two of four Z chromosomes in this species. We marked them as Z_3_ and Z_4_ chromosomes (Fig. 4, lower panel). To get more markers on Z_3_, we searched in the assembled genome sequence of *L. sinapis* (Talla et al. 2017) for a scaffold containing an ortholog of the *m5u-mt* gene located on *B. mori* chr. 8 (Fig. 4). We found that the scaffold_39 in the *L. sinapis* genome sequence consists not only of the *m5u-mt* gene ortholog but also of the ortholog of *Ctatpase* gene located on *B. mori* chr. 15 (Supplementary Table S2). This finding allowed us to select and test six BAC clones containing orthologs of *B. mori* chr. 15 genes (Supplementary Table S1). Three BAC probes of these six, 41J22, 17I6 and 19O6, mapped to the Z_3_ bivalent in *L. juvernica* males (Fig. 4a). We also confirmed that two and three BAC probes containing putative autosomal orthologs of *B. mori* chr. 8 and chr. 15 genes, respectively, mapped to autosomes in *L. juvernica* males (see the underlined genes in Fig. 4 and Supplementary Fig. S3c). Taken together, we conclude that the Z_3_ chromosome of *L. juvernica* consists of two segments, one corresponding to a part of chr. 8 and one to a part of chr. 15 in *B. mori*, and Z_4_ contains the other part corresponding to *B. mori* chr. 8.

All ten BAC probes that mapped to Z_3_ and Z_4_ chromosomes in *L. juvernica* mapped to each single bivalent in *L. sinapis* and *L. reali* males (Fig. 4b, c). The fact that both Z_3_ and Z_4_ of *L. juvernica* contain orthologs of *B. mori* chr. 8 indicates that they arose by fission (see ‘Discussion’). The order of ten BAC loci remained unchanged in *L. sinapis* and *L. reali* (Fig. 4b, c). We also confirmed that two representative BAC probes of these ten, 62A6 and 69P11, hybridised to one chromosome of the sex-chromosome multivalent in *L. sinapis* and *L. reali* females (Fig. 5c, e). This chromosome was designated as Z_3_ in *L. sinapis* and *L. reali*.

To confirm that the Z chromosomes shown in Figs. 2–4 are indeed different individual chromosomes, we performed BAC-FISH mapping of six representative probes in males of each *Leptidea* species. These six BAC probes mapped to four chromosomes (Z_1_–Z_4_) in *L. juvernica* (Fig. 5b), three chromosomes (Z_1_–Z_3_) in *L. sinapis* (Fig. 5d) and four chromosomes (Z_1_–Z_4_) in *L. reali* (Fig. 5f), respectively. Thus, we successfully identified all Z chromosomes in all three *Leptidea* species.

### Analysis of molecular differentiation in W chromosomes

**Fig. 6:**
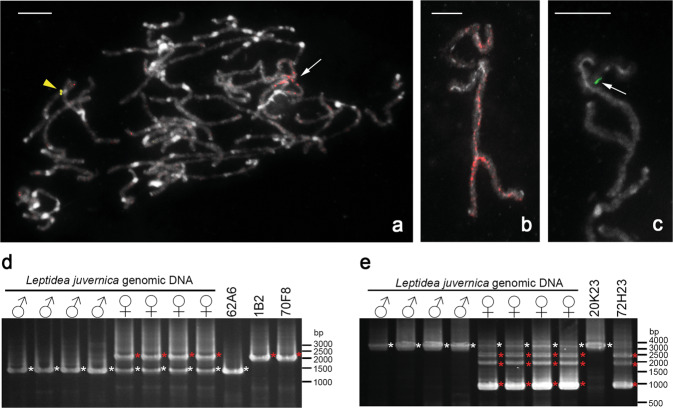
Analysis of W chromosomes in *Leptidea* species. **a**–**c** FISH with BAC probes derived from W chromosomes. Red, green and yellow represent hybridisation signals of BAC probes 1B2, 1J4 and 1A2, respectively. Chromosomes were stained with DAPI (grey). Bar = 10 μm. **a** Cy3-labelled 1B2 probe (red) painted part of the sex-chromosome multivalent (arrow) in female pachytene complement of *L. juvernica* and fluorescein-labelled 1A2 probe (yellow arrowhead) hybridised to an autosome bivalent. Sex-chromosome multivalents of *L. juvernica* showing painted segments by 1B2 probe (red) in three W chromosomes (**b**) and hybridisation signals of the 1J4 probe (green and arrow in **c**). **d** Gel showing PCR results using a primer set (Supplementary Table S1) designed based on the partial exon sequences of the *Leptidea* ortholog of the *B. mori Uch5l* gene. Genomic DNAs of both sexes and DNAs of BAC clones 62A6, 1B2 and 70F8 were used as templates. White and red asterisks indicate an amplified sequence of the *Uch5l* ortholog and a female-specific (W-linked) sequence of this ortholog, respectively. **e** Gel showing PCR results using a primer set (Supplementary Table S1) designed based on the partial exon sequences of the *Leptidea* ortholog of the *B. mori Gst8* gene. Genomic DNAs of both sexes and DNAs of BAC clones 20K23 and 72H23 were used as templates. White asterisks indicate an amplified sequence of the *Gst8* ortholog and red asterisks a female-specific (W-linked) sequence of this ortholog.

BAC clones derived from W chromosomes of *L. juvernica* were identified by FISH-based screening (Supplementary Text 2 and Supplementary Figs S1, S4). The W-BAC 1B2 probe painted part of the sex-chromosome multivalent in *L. juvernica* females (Fig. 6a, b) and another W-BAC clone 1J4 hybridised to a specific region of the sex-chromosome multivalent in *L. juvernica* females (Fig. 6c). The 1B2 probe also painted part of the sex-chromosome multivalent in other two *Leptidea* species, whereas hybridisation signals of the 1J4 probe were not detected in either of these species (Supplementary Fig. S5). Sequence analysis of sub-cloned W-BAC clones 1B2 and 1J4 and blast searches showed that most of the sub-cloned sequences were part of transposable elements, but two sub-clones (Nos 11 and 18) were part of the *Leptidea* ortholog of the *B. mori Uch5l* gene (Supplementary Table S3 and Supplementary Fig. S6a), which mapped to the Z_3_ chromosome in *L. juvernica* (Fig. 4). This result suggests that the two sub-cloned sequences are part of a Whomologue of the Z_3_-linked ortholog of the *Uch5l* gene.

We searched for W-specific sequences of the *Uch5l* ortholog by PCR with a set of designed primers (Supplementary Fig. S6a and Supplementary Table S1). PCR amplified two distinct fragments (1400 and 2095 bp) from female gDNA of *L. juvernica*, whereas only a single fragment was amplified from male gDNA (Fig. 6d). These PCR results also showed that the BAC clone 62A6, which mapped to the Z_3_ chromosome (Fig. 4), contains a common fragment of both sexes (Fig. 6d, white asterisks) and that the W-BAC clones 1B2 and 70F8, the latter identified by further BAC library screening, contain a female-specific fragment (Fig. 6d, red asterisks). The female-specific fragment (*Lj_Uch5l_W*) and the common fragment (*Lj_Uch5l_Z*) share sequences in their exons, but differ quite considerably in their introns (Supplementary Fig. S6b and Supplementary Table S4). The differences between introns are mainly due to randomly occurring insertions in the *Lj_Uch5l_W* intron (Supplementary Figs S6c, S7). The female-specific fragment was also amplified from gDNAs of *L. sinapis* and *L. reali* females and most of their sequences were well conserved in all three species, except for several insertions or deletions (Supplementary Fig. S8). We designed a primer set based on female-specific sequence in the intron of the *Uch5l* ortholog (Supplementary Table S5 and Supplementary Fig. S6b) to confirm the female-specific insertion. PCR with the female-specific primer set amplified a 341-bp fragment from female gDNAs of all three species (Supplementary Fig. S6c, asterisks), whereas no 341-bp fragments were detected in male gDNAs.

We found a sex-specific polymorphism in the *Leptidea* ortholog of the *Gst8* gene, located near the *Uch5l* gene on *B. mori* chr. 8 and we showed that the ortholog has a W-linked copy. PCR with a set of designed primers (Supplementary Fig. S9a and Supplementary Table S1) amplified a single fragment from male gDNA of *L. juvernica* (Fig. 6e, white asterisks), whereas several distinct fragments were amplified from female gDNA (Fig. 6e, red asterisks). We identified clone 20K23 containing a fragment of the *Gst8* ortholog that was common to both sexes (Fig. 6e). The 20K23 probe mapped to the same region of the Z_3_ chromosome in *L. juvernica* as the 62A6 probe containing the *Uch5l* ortholog (Supplementary Fig. S9b). These results suggest that the fragment amplified from both sexes is derived from the Z_3_ chromosome. In contrast, the BAC clone 72H23 contained several distinct female-specific fragments of the *Gst8* ortholog (Fig. 6e, red asterisks) and the 72H23 probe painted most of the W chromosomes in *L. juvernica* females (Supplementary Fig. S9c). Sequence analysis of the common fragment and the female-specific fragment of the *Gst8* ortholog revealed several female-specific (W-specific) deletions in the introns and the occurrence of at least three distinct copies of the *Gst8* ortholog on the *L. juvernica* W chromosomes (Supplementary Text 3 and Supplementary Fig. S9d).

**Fig. 7:**
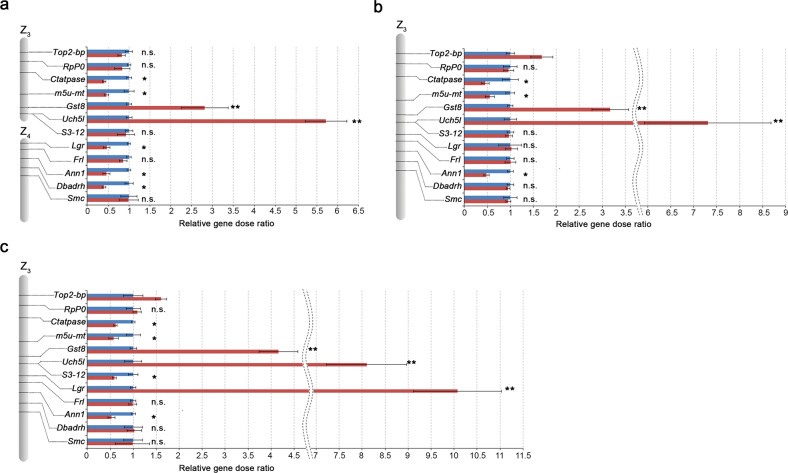
Female-to-male gene dose ratios of Z-linked genes in three *Leptidea* species. Results of qPCR showing female (red columns) to male (blue columns) relative dose ratios of Z_3_- and Z_4_-linked genes in *L. juvernica* (**a**) and Z_3_-linked genes in *L. sinapis* (**b**) and *L. reali* (**c**) normalised to the autosomal reference gene *RpS5* (average of target to reference gene dose in male = 1). Error bars represent SDs calculated from three independent samples (Supplementary Tables S6, S7 and S8). Double asterisks, asterisks and n.s. indicate a significant multiple difference (female-to-male > 2:1), a significant twofold difference (female-to-male = 1:2), and no significant difference (female-to-male = 1:1) in unpaired two-tailed *t* test for unequal variances, respectively (Supplementary Tables S6, S7 and S8).

**Fig. 8:**
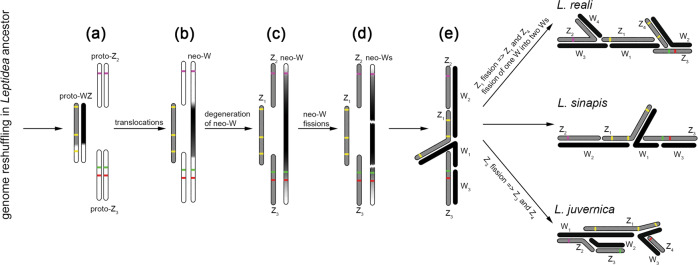
Hypothetical scenario of the multiple sex-chromosome evolution in three *Leptidea* species. This scenario is based on the results of BAC-FISH mapping presented in this study. The proposed steps in the evolution of multiple sex-chromosomes are shown in **a**–**e**. Proto-WZ indicates a putative pair of ancestral sex-chromosomes (W and Z), proto-Z_2_ and proto-Z_3_ two ancestral autosomes of the common ancestor of three *Leptidea* species. Positions of representative BAC clones for the respective Z chromosomes are indicated in yellow, purple, red and green as in Fig. 5.

To estimate the copy number of *Leptidea* orthologs of the *Uch5l* and *Gst8* genes on the W chromosome(s) and to assess if other Z_3_- and Z_4_-linked orthologs still remain covered on the W chromosome(s), we compared the relative gene dose of these orthologs between female and male gDNAs in the *Leptidea* species studied by qPCR (Fig. 7; Supplementary Fig. S10 and Supplementary Tables S6–S8). Because Z_1_- and Z_2_-linked orthologs have been identified as sex-linked genes by array-CGH in *L. juvernica* (Figs. 1–3), female-to-male relative gene dose ratios of these orthologs should be 1:2. Accordingly, we confirmed a twofold difference between the female and male gene dose in all tested Z_2_-linked orthologs by qPCR in all three species (Supplementary Fig. S10). Results of qPCR with Z_3_- and Z_4_-linked orthologs showed that the female-to-male relative gene dose ratios of the *Uch5l* and *Gst8* orthologs are ~5.7:1 and 2.8:1 in *L. juvernica* (Fig. 7a), 7.3:1 and 3.1:1 in *L. sinapis* (Fig. 7b) and 8:1 and 4.1:1 in *L. reali* (Fig. 7c), respectively. Female-to-male relative gene dose ratios of orthologs of the *Ctatpase*, *m5u-mt* and *Ann1* genes in all three species, *Lgr* and *Dbadrh* genes in *L. juvernica*, and the S3-12 gene in *L. reali* were 1:2 (Fig. 7 asterisks and Supplementary Tables S6–S8). In contrast, we found no significant difference between the female and male gene doses in orthologs of the *RpP0*, *Frl* and *Smc* genes in all three species, *Top2-bp* and *S3-12* genes in *L. juvernica*, *S3-12*, *Lgr* and *Dbadrh* genes in *L. sinapis*, and the *Dbadrh* gene in *L. reali* (Fig. 7 n.s. and Supplementary Tables S6–S8), suggesting that their orthologs also remained intact on the W chromosomes. Female-to-male relative gene dose ratios of the *Top2-bp* gene ortholog were 1:1 in *L. juvernica* but ~1.6:1 in *L. sinapis* and *L. reali*. Results of qPCR showed a tenfold difference between the female and male gene dose of the *Lgr* ortholog in *L. reali* (Fig. 7c).

## Discussion

In this work, we have deciphered the evolutionary origin of complex multiple sex-chromosomes in three cryptic *Leptidea* species. BAC-FISH mapping of Z-linked genes clearly showed that the multiple sex-chromosomes arose by several translocations and fusions between the ancestral WZ pair and autosomes, followed by several fissions (Figs. 2–4). The resulting 3–4 Z chromosomes are composed each of 2–3 conserved synteny blocks, in which the gene order remained well conserved in all three species. However, the Z chromosomes in individual species were differentiated by further rearrangements, either by fusion or fission (Fig. 5). In accordance with phylogenetic relationships between the three species (Dincă et al. 2011; Lukhtanov et al. 2011; Šíchová et al. 2015; Fig. 5 in this study), the Z_1_ of *L. juvernica* and *L. sinapis* most likely split into two Z chromosomes (Z_1_ and Z_4_) in *L. reali* after *L. reali* and *L. sinapis* diverged from their common ancestor because both Z_1_ and Z_4_ of *L. reali* contain genes of the original Z-chromosome in the ancestral lepidopteran karyotype (Van’t Hof et al. 2013; Ahola et al. 2014). The Z_3_ of *L. sinapis* and *L. reali* corresponds to Z_3_ and Z_4_ chromosomes in *L. juvernica* (Fig. 4). Since both Z_3_ and Z_4_ of *L. juvernica* contain orthologs of *B. mori* chr. 8 genes that are located on one autosome in the ancestral lepidopteran karyotype, the two Z chromosomes most likely originated by fission of Z_3_ chromosome after *L. juvernica* diverged from *L. sinapis* plus *L. reali*. These findings strongly suggest that the sex-chromosome constitution in the common ancestor of these three *Leptidea* species was similar to that of *L. sinapis* (Fig. 5).

Conserved synteny blocks and conserved order of genes in the multiple Z chromosomes of *Leptidea* species studied are in line with current knowledge of the genome architecture in Lepidoptera. Most lepidopteran species have a chromosome number equal to or near the ancestral number of *n* = 31, and synteny of genes, including their order, is relatively well conserved even among distant species (Van’t Hof et al. 2013; Ahola et al. 2014; Yasukochi et al. 2016). The model species for Lepidoptera, *B. mori*, has a haploid chromosome number of *n* = 28 and its karyotype evolved by three chromosome fusions from the ancestral *n* = 31, except for a few inversions and translocations (Van’t Hof et al. 2013; Ahola et al. 2014; Yasukochi et al. 2016). A similar explanation applies to the karyotype evolution in other lepidopteran species with reduced chromosome numbers (Pringle et al. 2007; Yasukochi et al. 2009; Yoshido et al. 2011b). Our results, together with earlier reported variation in chromosome numbers in the genus *Leptidea* (Dincă et al. 2011; Lukhtanov et al. 2011; Šíchová et al. 2015, 2016), suggest dynamic genome rearrangements that occurred not only in sex chromosomes but also in autosomes during karyotype evolution before *Leptidea* species diverged from a common ancestor. Consequently, the *Leptidea* species do not exhibit a conserved macrosynteny of genes compared with the putative ancestral karyotype of Lepidoptera (Van’t Hof et al. 2013; Ahola et al. 2014). The recent genome assembly of another pierid butterfly, *Pieris napi*, showed conserved microsynteny blocks of genes in autosomes but broken macrosynteny blocks, also suggesting dynamic genome rearrangements (Hill et al. 2019).

Based on the above findings and our results, we proposed a hypothetical scenario of the evolution of multiple sex-chromosomes in the three *Leptidea* species studied (Fig. 8). Given the similar constitution of sex chromosomes, we infer that major genome reshuffling occurred in the common ancestor of these *Leptidea* species. As a result, proto-WZ chromosomes in ancestral females originated by translocation of a part of the autosome orthologous to *B. mori* chr. 17 (Fig. 8a, white parts of proto-WZ) onto the ancestral pair of WZ sex-chromosomes and two pairs of autosomes, referred to as proto-Z_2_ and proto-Z_3_, arose by fusion of several synteny blocks of the ancestral lepidopteran karyotype (Fig. 8a). At that time, ancestral males already had the current structure of Z_1_, Z_2_ and Z_3_ chromosomes as shown in Fig. 5d, d´ for *L. sinapis*, but proto-Z_2_ and proto-Z_3_ were still autosomes in both sexes. In the next step, proto-Z_2_ and proto-Z_3_ were translocated onto the proto-W chromosome and their unfused homologues thus turned from autosomes to Z_2_ and Z_3_ sex-chromosomes (Fig. 8b). In the resulting neo-W chromosome, the originally autosomal parts gradually degraded due to the absence of recombination in female meiosis (Fig. 8c). According to the degeneration of genes on the neo-W chromosomes demonstrated by the qPCR results (Fig. 7 and Supplementary Fig. S10), the proto-Z_2_ translocation onto the proto-W chromosome is most probably older than the proto-Z_3_ translocation. In the final step, two fissions occurred in the neo-W (Fig. 8d), resulting in a multiple sex-chromosome constitution with three W (W_1–3_) and three Z (Z_1–3_) chromosomes, as found in *L. sinapis* females (Fig. 8e). Subsequently, during the process of speciation, several further rearrangements of sex chromosomes contributed to the differentiation of closely related species. Fission of one of the W chromosomes and fission of the Z_1_ chromosome led to the W_1–4_Z_1–4_ constitution, as found in *L. reali*, whereas the Z_3_ fission resulted in the W_1–3_Z_1–4_ constitution, as found in *L. juvernica* (Fig. 8e). In this work, we showed that most homologues of Z_2_-linked genes decayed in the W chromosomes of all three species (Supplementary Fig. S10), while some homologues of Z_3_- and Z_4_-linked genes are still preserved (Fig. 7). However, it can be expected that degeneration of the originally autosomal parts of the W chromosomes, corresponding to Z_3_ and Z_4_ chromosomes, will proceed through the accumulation of transposable elements, duplications, insertions and/or deletions (Fig. 6d–e; Supplementary Figs S6–S9 and Supplementary Table S3). Especially qPCR results strongly suggest that two *Uch5l* and *Gst8* orthologs in all three species, and thus in their common ancestor, and *Lgr* ortholog in *L. reali* were amplified on the W chromosome(s) during the W chromosome degeneration (Fig. 7, double asterisks; cf. Bachtrog et al. 2019). Nevertheless, the finding of well-preserved homologues of genes of autosomal origin in the W chromosomes supports the proposed hypothetical scenario of evolution of multiple sex-chromosomes in *Leptidea* species.

In Lepidoptera, the correspondence of ‘ancestral’ Z-linked genes is highly conserved across the phylogenetic tree (Beldade et al. 2009; Yasukochi et al. 2009; Nguyen et al. 2013; Van’t Hof et al. 2013; Dalíková et al. 2017a; Fraïsse et al. 2017), but the gene order may be changed by intrachromosomal rearrangements (Yasukochi et al. 2009; Van’t Hof et al. 2013). A similar picture emerged in this study for *Leptidea* species. Despite many chromosomal rearrangements in their genomes, most ‘ancestral’ Z-linked genes mapped to the Z_1_ (and Z_4_ in *L. reali*) chromosome, except for a few genes (maximum 9%; see array-CGH results in Fig. 1b), but the gene order of ancestral Z-linked genes was changed compared with the Z chromosome of *B. mori* (Fig. 2). Lepidopteran Z chromosomes are known to play an important role in sex determination (Kiuchi et al. 2014), adaptation and speciation (Presgraves 2002; Dopman et al. 2005). The fact that the synteny block of the ‘ancestral’ Z-linked genes escaped the dynamic reorganisation of *Leptidea* genomes and remained conserved is consistent with the key role of Z chromosomes in the evolution of Lepidoptera.

In most lepidopteran species, the maternally inherited W chromosome is composed of heterochromatin and, due to the absence of recombination, is characterised by extensive genetic erosion and accumulation of repetitive DNA sequences (Abe et al. 2005; Fuková et al. 2007; Marec et al. 2010; Yoshido et al. 2016; Dalíková et al. 2017b). Evolutionary mechanisms of this genetic erosion have been demonstrated in the Y chromosomes of species with male heterogamety, especially in the *Drosophila* genus (Bachtrog 2005, 2013; Charlesworth et al. 2005). In evolutionary old and well-differentiated W chromosomes, homologous sequences of the ‘ancestral’ Z-linked genes were rarely found (Gotter et al. 1999; Van’t Hof et al. 2013). The multiple W chromosomes in three *Leptidea* species represent in fact evolutionary strata (Wright et al. 2016) of different ages and levels of differentiation that contain each a segment of autosomal origin (Fig. 8). Taking into account the estimated divergence times, which range from 1–2 million years in *L. sinapis* vs. *L. reali* to 2.5–3.5 million years in *L. juvernica* vs. the two other species (Talla et al. 2017, 2019a), these W chromosomes can be considered evolutionarily young and allow us to compare the level of their differentiation. We identified W homologues of Z_3_- and Z_4_-linked genes in all three species with some evidence of ongoing molecular differentiation, such as insertions, deletions and duplications (Fig. 6 and Supplementary Figs S6–S9). Moreover, about half of the examined Z_3_- and Z_4_-linked genes still had their homologues on the neo-W chromosomes in all three species (Fig. 7). The level of differentiation of these neo-W chromosomes thus roughly corresponds to the differentiation of the 1 million years old *Drosophila miranda* neo-Y chromosome, in which more than 40% of the genes initially present on the ancestral neo-Y chromosome became pseudogenes or was completely lost (Bachtrog 2013). However, we found no evidence of W homologues of Z_1_- and Z_2_-linked genes, suggesting advanced molecular differentiation of the relevant W chromosomes (Supplementary Fig. S10) comparable to the neo-W chromosome of *Danaus* species, which is believed to be older than 5 million years (Mongue et al. 2017). A relatively young neo-W chromosome with incomplete degeneration of the originally autosomal segment was also described in a wild silkmoth, *S. cynthia walkeri* (Yoshido et al. 2016). On the contrary, in evolutionary old neo-sex chromosomes that arose by fusion of ancestral sex-chromosomes with an autosome, it is difficult to demonstrate the neo-W chromosome due to progressive degeneration and the absence of homology (Mongue et al. 2017; Picq et al. 2018; Carabajal Paladino et al. 2019).

We reconstructed the evolution of multiple sex-chromosomes in three cryptic *Leptidea* species and identified species-specific differences in their composition. Could differences in sex-chromosome constitutions between these species have contributed to their speciation? Our results suggest that multiple sex-chromosome systems predated the formation of *Leptidea* species studied. Thus, the initial sex-chromosome turnover could not have played a role in reproductive isolation between these species. However, subsequent structural rearrangements leading to a species-specific number of multiple sex-chromosomes, namely fissions of several neo-sex chromosomes, could have significantly contributed to reproductive isolation due to mis-segregation of these neo-sex chromosomes in hybrids. In addition, different rates of neo-W chromosome degeneration (Fig. 7) could have also contributed to the divergence of *Leptidea* populations (Filatov 2018). A recent analysis of the whole-genome sequence data did not find any traces of post-divergence gene flow between these species (Talla et al. 2019a). These results suggest well-established reproductive barriers between these species, which include strong pre-mating reproductive isolation because females only accept conspecific males (Friberg et al. 2008; Dincă et al. 2013). The three *Leptidea* species showed a considerably lower level of genome-wide diversity than most other butterflies examined, indicating reduced effective population sizes (Talla et al. 2019b). They also showed a significantly reduced genetic diversity on the ‘ancestral’ Z-linked genes (corresponding to Z_1_ in this study, multiple Z-chromosomes were not considered) and a significantly higher level of genetic differentiation on the ‘ancestral’ Z-linked genes in comparison with the ‘ancestral’ autosomal genes (Talla et al. 2019a). Especially the latter results are consistent with the so-called ‘Faster-Z effect’, which means that the sex-linked genes are subjected to a faster rate of evolution (Mank et al. 2010). In *Leptidea* species, we showed that different sets of originally autosomal genes have become Z-linked. This greatly increased the number of Z-linked genes, which could significantly accelerate the accumulation of genetic incompatibilities between populations, thus contributing to their divergence and subsequent speciation (cf. Turelli and Begun 1997).

## Supplementary information

Supplementary Information

## Data Availability

The raw reads generated in this study have been deposited in the NCBI Sequence Read Archive (SRA) database under the accession number SRR10381488 (Bioproject PRJNA586890). Other datasets generated in this study are available in the Dryad repository (10.5061/dryad.h70rxwddw). The custom Python script used for analysis of array-CGH data is available at https://github.com/avolenikova/aCGH_scripts.
