# Supplementary material for: Evolution of multiple sex-chromosomes associated with dynamic genome reshuffling in Leptidea wood-white butterflies
Source: Heredity (Edinb). 2020 Jun 9;125(3):138–54. doi: 10.1038/s41437-020-0325-9 (PMC7426936; doi:10.1038/s41437-020-0325-9)
Supplement: Supplementary file 1 — Supplementary Information [file 41437_2020_325_MOESM1_ESM.pdf]

Supplementary Information for

Evolution of multiple sex chromosomes associated with dynamic genome reshuffling in *Leptidea* wood white butterflies

Atsuo Yoshido, Jindra Šíchová, Kristýna Pospíšilová, Petr Nguyen, Anna Voleníková, Jan Šafář, Jan Provazník, Roger Vila, František Marec*

*Corresponding author: marec@entu.cas.cz

The PDF file includes:

Supplementary Methods
Supplementary Text 1
Supplementary Text 2
Supplementary Text 3
Fig. S1
Fig. S2
Fig. S3
Fig. S4
Fig. S5
Fig. S6
Fig. S7
Fig. S8
Fig. S9
Fig. S10
Table S1
Table S2
Table S3
Table S4
Table S5
Table S6
Table S7
Table S8

Supplementary Methods

Array-CGH analysis in *Leptidea juvernica*. First we generated about 248 million 100-bp paired-end reads from genes expressed in a female larva. The raw reads were deposited in the NCBI Sequence Read Archive (SRA) database under the accession number SRR10381488 (Bioproject PRJNA586890). About 93% of the pairs that remained after quality filtering were assembled de novo by SOAPdenovo and Trinity assemblers. The resulting assemblies were merged and redundant transcripts were reduced by the EvidentialGene pipeline (Gilbert 2013). The pipeline produced a dataset of 30057 transcripts, which was further used for the HaMStR search for transcripts orthologous to the Lepidoptera core ortholog set constructed by Kawahara and Breinholt (2014). Second we searched for *Leptidea* orthologs of *Bombyx mori* genes in the EvidentialGene output using HaMStR (Ebersberger et al. 2009) with the '-representative' option and the Lepidoptera core ortholog set constructed. The search identified 4398 orthologous sequences, which were then used to design the Agilent custom microarray containing 37909 probes. The *Leptidea* orthologs were used to design 60-mer oligonucleotide probes for custom-made microarray slides using Agilent Technologies eArray design wizard (https://earray.chem.agilent.com/earray/). Female and male genomic DNAs (gDNAs) were extracted from 5th instar larvae using CTAB protocol (Winnepenninckx et al. 1993). DNA samples were quantified using the Qubit dsDNA BR Assay kit (Thermo Fisher Scientific, Waltham, MA, USA) and labelled. DNA digestion, labelling, and array-CGH were performed by GenLabs (Prague, Czech Republic) following a protocol for Agilent oligonucleotide array-based CGH for genomic DNA analysis. Hybridization intensities were extracted using Agilent's Feature Extraction software. Filtering and analysis of feature intensities followed Baker and Wilkinson (2010) and were implemented in the Python script (https://github.com/avolenikova/aCGH_scripts). By default, the cut-off value of 0.5 was used to identify Z-linked genes. The non-redundant transcripts, list of the *L. juvernica* orthologs, array design, and array-CGH results are available in the Dryad repository (doi:10.5061/dryad.h70rxwdd).

BAC-FISH mapping. For chromosome preparations, the tissues (ovaries, testes, and wing imaginal discs) were dissected in saline solution, swollen in a hypotonic solution (75 mM KCl) for 10–15 min (wing discs and testes always, ovaries optionally), and then fixed in Carnoy's fixative solution (ethanol, chloroform, acetic acid; 6:3:1) for 10–20 min. Cells were dissociated in 60% acetic acid and spread on a heating plate at 45°C. All chromosome preparations were passed through a graded ethanol series (70%, 80%, and 100%) and stored at –80°C.

BAC-DNA was extracted using the Plasmid Midi Kit (Qiagen, Hilden, Germany) or the NucleoBond Xtra Midi plasmid purification kit (Macherey-Nagel, Düren, Germany). DNA labelling was performed by nick translation using a mixture of DNase I and DNA polymerase I (both Thermo Fisher Scientific). The 50 µl reaction contained 1 µg of BAC DNA; 25 µM dATP, dCTP, and dGTP; 17 µM dTTP; 8 µM labelled nucleotides with either aminoallyl-dUTP-Cy3 or fluorescein-12-dUTP (both Jena Bioscience, Jena, Germany); 1× DNA polymerase I buffer and 5 µl of the above enzyme mix. The reaction was incubated at 15°C for 5 h.

Chromosome preparations were removed from the freezer, dehydrated in the graded ethanol series, and air-dried. Chromosomes were denatured in 70% formamide (Sigma-Aldrich, St. Louis, MO, USA) in 2× SSC for 3.5 min at 68–70°C. For one preparation, we used a probe cocktail containing 100–500 ng of each labelled DNA, 3–10 µg of unlabelled sonicated male gDNA of the respective species (extracted from larvae by standard phenol-chloroform procedure), and 25 µg of sonicated salmon sperm DNA (Sigma-Aldrich) in 10 µl hybridization buffer of 50% formamide, 10% dextran sulphate (Sigma-Aldrich) in 2× SSC. The probe cocktail was denatured at 90°C for 5 min and then hybridized to denatured chromosomes at 37°C for 3 days. Then the slides were washed at 62°C for 5 min in 0.1× SSC with 1% Triton X-100) and counterstained and mounted in antifade based on DABCO (1,4-diazabicyclo (2.2.2)-octane), containing 0.5 µg/ml DAPI (4',6-diamidino-2-phenylindole) (both Sigma-Aldrich). Preparations were observed in a Zeiss Axioplan 2 microscope (Carl Zeiss, Jena, Germany). Digital images were captured with an Olympus CCD monochrome camera XM10 equipped with cellSens 1.9 digital imaging software (Olympus Europa Holding, Hamburg, Germany) and processed with Adobe Photoshop CS4 (Adobe Systems Inc., San Jose, CA, USA) as follows. The images were pseudo-coloured and superimposed and intensities of hybridization signals were adjusted using Brightness/Contrast in Adobe Photoshop.

Analysis of W-homologs of Z-linked orthologs. Genomic DNAs were extracted separately from females and males in three *Leptidea* species by standard phenol-chloroform procedure. For further analysis, we designed primers (Supplementary Table S1) based on sequences of *Leptidea* orthologs of *B. mori Uch5l* and *Gst8* genes, which were identified in BAC clones derived from *L. juvernica* W chromosomes (see main text, Results). PCR amplifications of these orthologs were performed using a reaction mixture composed of 10 ng of template gDNA, 10 pmol of each primer, 0.25 U of Ex-*Taq* polymerase, and 1.0 µl of 10× Ex-*Taq* buffer (Takara, Otsu, Japan). The PCR consisted of initial denaturation for 5 min at 94°C, followed by 35 cycles of denaturation for 30 s at 94°C, annealing for 30 s at 55°C for *Gst8* or 60°C for *Uch5l*, elongation for 180 s at 72°C, and final elongation for 5 min at 72°C. For the detection of female-specific fragments of

the *Uch5l* gene in three *Leptidea* species, we designed a primer set (Supplementary Table S5) based on female-specific sequences (*Uch5l_W*) conserved in these species (see main text, Results). In this case, the PCR profile was modified as follows: initial denaturation for 5 min 94°C, 30 cycles of denaturation for 30 s at 94°C, annealing for 30 s at 60°C, and elongation for 30 s at 72°C and a final extension step for 1 min at 72°C. Primers for the *Leptidea* ortholog of the *B. mori RpS5* gene were used as positive controls (Supplementary Table S1).

References

Baker RH, Wilkinson GS (2010) Comparative Genomic Hybridization (CGH) reveals a neo-X chromosome and biased gene movement in stalk-eyed flies (genus Teleopsis). PLoS Genet. 6: e1001121.

Ebersberger I, Strauss S, von Haeseler A (2009) HaMStR: Profile hidden Markov model based search for orthologs in ESTs. BMC Evol Biol 9: 157.

Gilbert D (2013) Gene-omes built from mRNA seq not genome DNA. 7th Annual Arthropod Genomics Symposium. Notre Dame. F1000Research 5: 1695. doi: 10.7490/f1000research.1112594.1.

Kawahara AY, Breinholt JW (2014) Phylogenomics provides strong evidence for relationships of butterflies and moths. Proc Biol Sci 281: 20140970.

Winnepenninckx B, Backeljau T, De Wachter R (1993) Extraction of high molecular weight DNA from molluscs. Trends Genet 9: 407.

Supplementary Text 1. Gene movement out of the *Leptidea juvernica* Z_1 chromosome

Some BAC probes containing *Leptidea* orthologs of *B. mori* Z-linked genes did not map to Z chromosomes in three *Leptidea* species. The 62O17 probe containing the ortholog of the *Pgd* gene provided discrete hybridization signals in a single autosomal bivalent (Supplementary Fig. S2b). By PCR screening with primers designed on exon sequences, we found that three BAC clones (91C3, 96D19, and 66L7) contain each different size-fragment of a *Leptidea* ortholog of the *B. mori Tpi* gene (Supplementary Table S1). Sequence analysis of these distinct fragments of the *Tpi* ortholog amplified by PCR from one *L. juvernica* specimen showed that they contained an intron and the differences in fragment size resulted from polymorphisms in intron length. Hybridization signals of those BAC 96D19 and 66L7 probes co-localized in an autosomal bivalent and the BAC 91C3 probe mapped to another autosomal bivalent (Supplementary Fig. S2c). These results suggest orthologs of *Pgd* and *Tpi* are not Z-linked genes in *Leptidea juvernica*.

Supplementary Text 2. Identification of BAC clones derived from W chromosomes of *Leptidea juvernica*

To identify BAC clones derived from W chromosome of *Leptidea juvernica*, we screened the BAC library using FISH-based screening (Supplementary Fig. S1). Randomly selected 64 BAC clones were separately cultured in 5.0 mL LB medium with chloramphenicol. Cultures of eight BAC clones were pooled and then their pooled DNAs were extracted as described above. The eight-BAC-DNA pools were labelled with either aminoallyl-dUTP-Cy3 or fluorescein-12-dUTP (both Jena Bioscience) by nick translation and hybridized to female pachytene chromosomes of *L. juvernica*. If the eight-BAC probe hybridized to any W chromosome, BAC-DNA was extracted separately from each of the eight BAC clones, and then the respective BAC probes were hybridized to female pachytene chromosomes.

By FISH-based screening of 64 BAC clones, two BAC clones (1B2 and 1J4) were identified as candidates for W-derived clones. We also selected one autosome-derived BAC clone (1A2) and used it as a positive control when mapping W-derived BAC clones by FISH. The 1B2 probe painted part of the sex chromosome multivalent in *L. juvernica* females (see main text, Fig. 6a, b) and strong hybridization signals were also observed in female interphase nuclei (Supplementary Fig. S4a). In male interphase nuclei, clear hybridization signals of the autosome-derived BAC 1A2 probe (see main text, Fig. 6a, arrowhead) were detected (Supplementary Fig. S4b, arrowheads), whereas signals of the 1B2 probe were very weak and scattered. Another candidate W-BAC clone 1J4 hybridized to a specific region of the sex chromosome multivalent in *L. juvernica* females (see main text, Fig. 6c), and strong signals of the 1J4 probe were also observed in female interphase nuclei but not in male nuclei (Supplementary Fig. S4c, d). These results suggest that both BAC 1B2 and 1J4 clones are derived from the W chromosomes of *L. juvernica*. In addition, BAC-FISH on female mitotic chromosomes of *L. juvernica* showed that the 1B2 probe highlighted either most or part of all three W chromosomes, while the 1J4 probe hybridized only to the terminal part of a single W chromosome (Supplementary Fig. S4e, f).

Supplementary Text 3. Sex-specific polymorphism in the *Leptidea juvernica* ortholog of the *Gst8* gene

Both common and female-specific fragments of the *Gst8* ortholog were cloned and sequenced. The results showed that *L. juvernica* females have a common fragment, *Lj_Gst8_Z*, which is derived from the Z_3 chromosome, and three female-specific fragments, *Lj_Gst8_W*^{a} (899 bp), *Lj_Gst8_W*^{b} (954 bp), and *Lj_Gst8_W*^{c} (2502 bp) (Supplementary Fig. S9a; Supplementary Table S4). However, female-specific fragments of about 2000 bp shown in Fig. 6e were missing. Here we demonstrate that these 2000 bp fragments are heteroduplexes formed by the *Lj_Gst8_W*^{c} fragment and either *Lj_Gst8_W*^{a} or *Lj_Gst8_W*^{b} fragments. (1) Four fragments (*Lj_Gst8_Z* and *Lj_Gst8_W*$^{a\text{-}c}$) were separately amplified with a primer set for *Gst8* (Supplementary Table S1) from the respective cloned fragments (see lanes L1–4 below). (2) Several PCR products were mixed (all fragments and only female specific fragments), denatured at 95°C for 5 min and then cooled at 4°C for 2 min. (3) Mixed, denatured and cooled PCR products in step 2 were run on a 1% agarose gel stained with ethidium bromide.

In L5 and L7 of mixed, denatured and cooled PCR products, fragments of about 2000 bp were observed similar to PCR products from W-BAC clone, 72H23 and female gDNA (see asterisks in lanes L5–8 below). Since only female-specific fragments (L2 + L3 + L4) were mixed in L5, fragments of about 2000 bp should be heteroduplexes formed by the *Lj_Gst8_W*^{c} fragment and either *Lj_Gst8_W*^{a} or *Lj_Gst8_W*^{b} fragments.

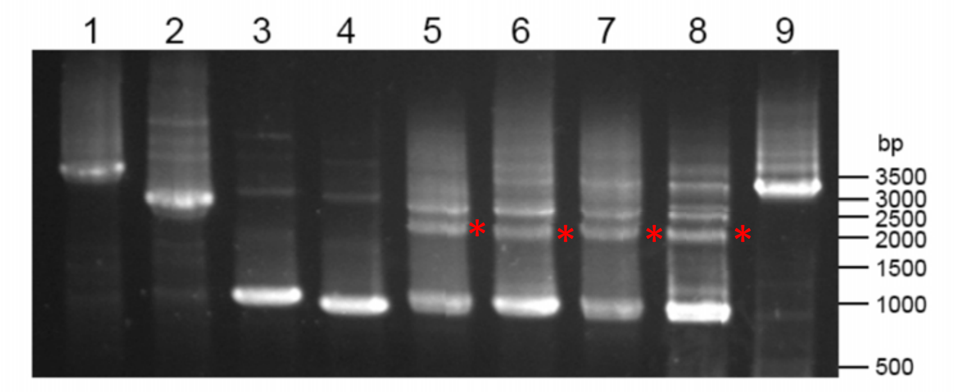

L1: PCR product amplified from cloned *Lj_Gst8_Z* (3577 bp)
L2: PCR product amplified from cloned *Lj_Gst8_W*^{C} (2502 bp)
L3: PCR product amplified from cloned *Lj_Gst8_W*^{b} (954 bp)
L4: PCR product amplified from cloned *Lj_Gst8_W*^{a} (899 bp)
L5: Mixed, denatured and cooled PCR products (L2 + L3 + L4)
L6: PCR product amplified from BAC 72H23 clone
L7: Mixed, denatured and cooled PCR products (L1 + L2 + L3 + L4)
L8: PCR product amplified from female gDNA
L9: PCR product amplified from male gDNA

By above experiment, we confirmed the female-specific fragments of about 2000 bp shown in the main text (Fig. 6e) were heteroduplexes formed by the *Lj_Gst8_W*^{c} fragment and either *Lj_Gst8_W*^{a} or *Lj_Gst8_W*^{b} fragments. Comparison of their sequences revealed female-specific (W-specific) deletions in the introns of *Lj_Gst8_W*^{b} and *Lj_Gst8_W*^{c} fragments and deletions not only in the intron but also in the exon of *Lj_Gst8_W*^{a} fragment (Supplementary Fig. S9a). The *Lj_Gst8_W*^{a} fragment thus encodes a truncated protein (Supplementary Fig. S9d).

- Separately cultivate 64 individual BAC clones
- Mix 8 BAC clones to make BAC-DNA pools for screening (see below)
- Extract BAC-DNA from each pool
- Prepare fluorochrome-labelled probe from each BAC-DNA pool
- Test each pool-probe by BAC-FISH

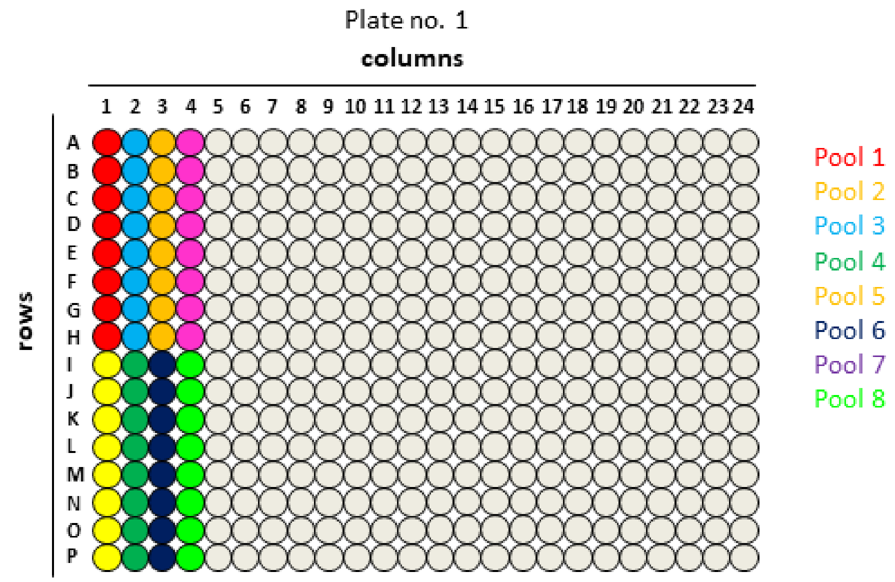

Fig. S1. Flowchart of FISH-based screening for identification of BAC clones derived from W chromosomes of *Leptidea juvernica*.

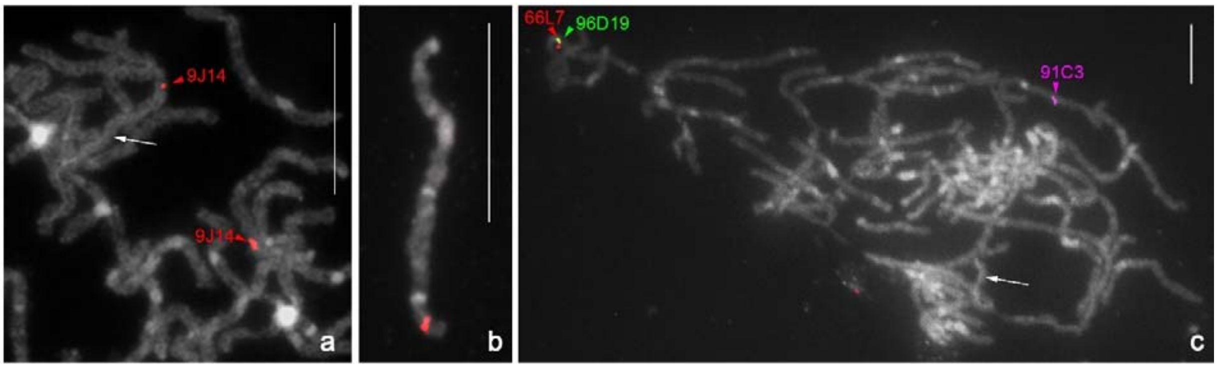

Fig. S2. FISH mapping of BAC probes containing *Leptidea* orthologs of *B. mori Prm*, *Pgd* and *Tpi* genes to female pachytene chromosomes of *L. juvernica*. Chromosomes were stained with DAPI (grey). Bar = 10 µm. Arrow indicates the sex chromosome multivalent of *L. juvernica* female. (**a**) A BAC 9J14 probe (red signals) containing the *Prm* ortholog mapped to not only sex chromosome multivalent but also an autosomal bivalent. (**b**) A BAC 62O17 probe (red signals) containing the *Pgd* ortholog mapped to an autosomal bivalent. (**c**) Three BAC clones (91C3, 96D19, and 66L7) contain each different size-fragment of the *Tpi* ortholog (see Supplementary Table S1). Hybridization signals of the 96D19 (green) and 66L7 (red) probes co-localized in an autosomal bivalent and the 91C3 probe (purple signals) mapped to another autosomal bivalent in female pachytene chromosomes.

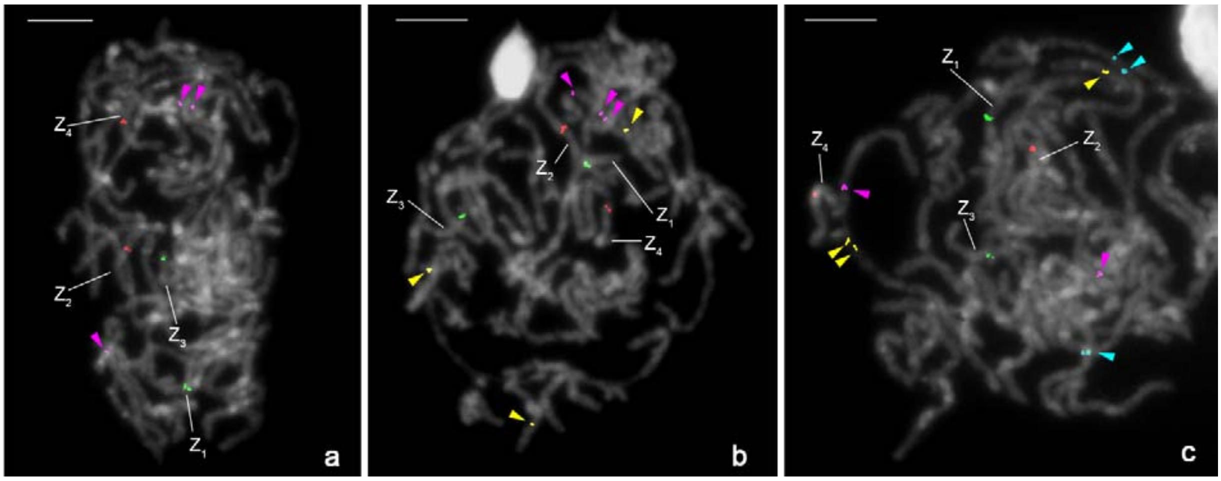

Fig. S3. FISH mapping of Z chromosome-derived and autosome-derived BAC probes in male pachytene chromosomes of *Leptidea juvernica*. Chromosomes were stained with DAPI (grey). Bar = 10 µm. Representative BAC probes, 92J7 (green signals) for Z_1, 94J6 (red signals) for Z_2, 62A6 (green signals) for Z_3, and 69P11 (red signals) for Z_4, were used to distinguish respective Z chromosomes. Z_1 versus Z_3 and Z_2 versus Z_4 were easily distinguished by differences in length of these chromosomes. Arrowheads indicate signals of tested BAC probes. (**a**) Three BAC probes, 66E20, 65E15, and 93F8 (purple signals), containing *Leptidea* orthologs of *B. mori* chr. 17 genes mapped to autosomal bivalents. (**b**) Three BAC probes, 95K24, 43I3, and 43L15 (yellow signals), containing *Leptidea* orthologs of *B. mori* chr. 11 genes and three BAC probes, 49I8, 94G12, and 49E5 (purple signals), containing *Leptidea* orthologs of *B. mori* chr. 24 genes mapped to autosomal bivalents. (**c**) Three BAC probes, 95N3, 41B20, and 42F24 (light blue signals), containing *Leptidea* orthologs of *B. mori* chr. 7 genes, two BAC probes, 95D23 and 70D8 (purple signals), containing orthologs of *B. mori* chr. 8 genes and three BAC probes, 9M16, 96L18, and 89C2 (yellow signals), containing orthologs of *B. mori* chr. 15 genes mapped to autosomal bivalents.

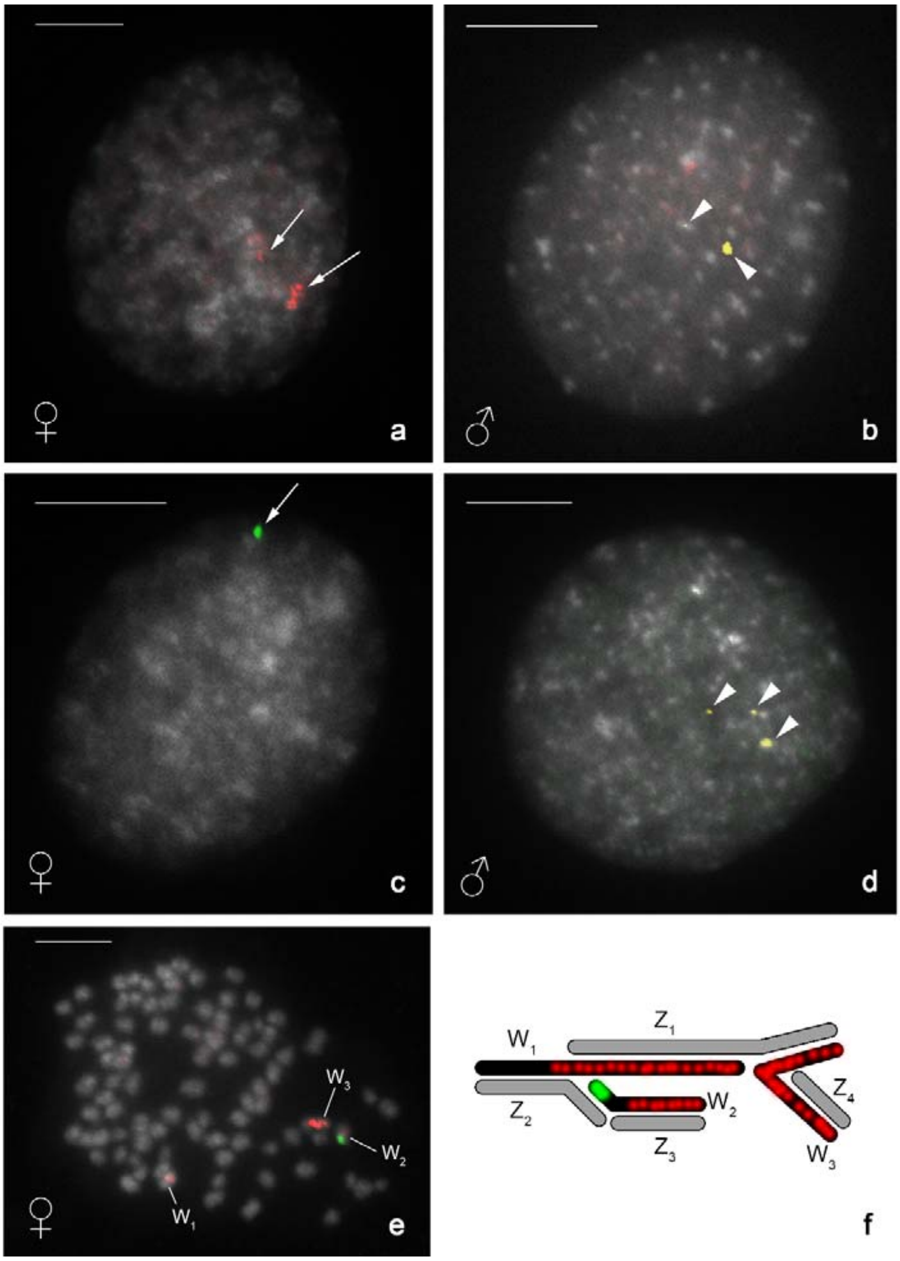

Fig. S4. FISH with BAC probes derived from W chromosomes of *Leptidea juvernica*. Nuclei and chromosomes were stained with DAPI (grey). Bar = 10.0 µm. Red, green, and yellow indicate hybridization signals of BAC 1B2, 1J4, and 1A2 probes. (**a** and **c**) Interphase nucleus of *L. juvernica* female. Arrows indicate hybridization signals of W-derived BAC probes. (**b** and **d**) Interphase nucleus of *L. juvernica* male. Arrowheads indicate hybridization signals of the BAC probe 1A2 (the 1A2 BAC clone was identified by FISH-based screening) derived from an autosome (Fig. 6a, arrowhead), which was used as a positive control. (**e**) Mitotic metaphase of *L. juvernica* female with all three W chromosomes (W_1-W_3) identified by BAC probes. (**f**) Schematic illustration of multiple sex chromosomes of *L. juvernica* females based on FISH with BAC probes derived from W chromosomes. Grey and black indicate Z and W chromosomes, respectively.

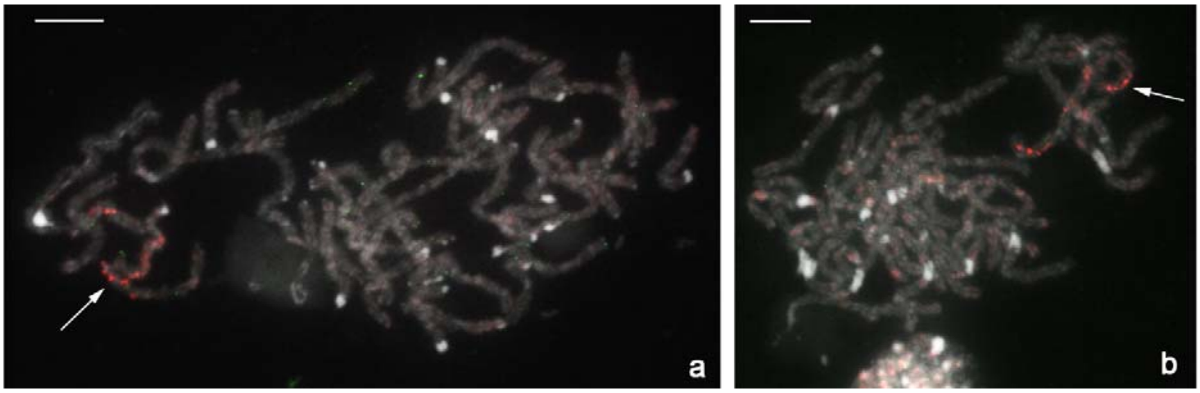

Fig. S5. Cross-hybridization of BAC probes derived from *L. juvernica* W chromosomes. Chromosomes were stained with DAPI (grey). Bar = 10.0 µm. The 1B2 probe (red) painted part of the sex chromosome multivalents in female pachytene complements of *L. sinapis* (**a**) and *L. reali* (**b**), but no hybridization signals of the 1J4 probe (green) were detected.

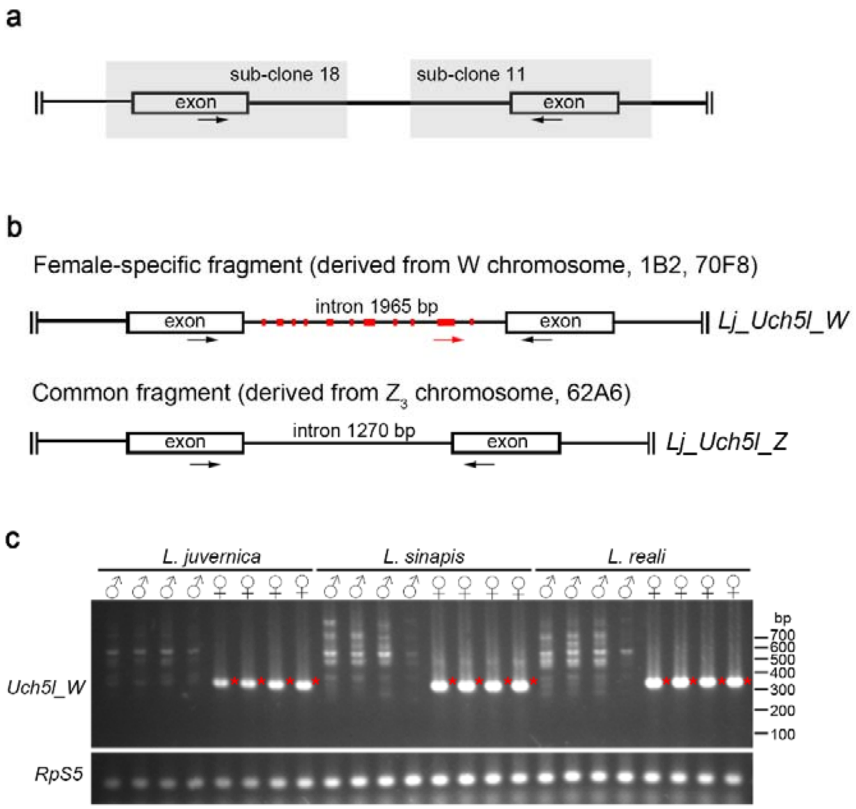

Fig. S6. Identification and characterization of a W-specific orthologous sequence of the *B. mori Uch5l* gene in *Leptidea juvernica*. (**a**) Schematic illustration of sequences Nos 11 and 18 (grey boxes) obtained by sub-cloning of the BAC clone 1B2, which is derived from *L. juvernica* W chromosomes. Arrows indicate the positions of designed primers. (**b**) Schematic illustrations of partial sequences of *Leptidea* orthologs of the *B. mori Uch5l* gene. Female-specific fragment (upper scheme, *Lj_Uch5l_W*) was obtained from genomic DNA of females and DNA of the 1B2 BAC clone, common fragment (lower scheme, *Lj_Uch5l_Z*) from genomic DNAs of both sexes and DNA of the 62A6 BAC clone. Small red boxes indicate insertions in the intron of the female-specific fragment and arrows the positions of designed primers. (**c**) Gel showing PCR results using a primer set (Supplementary Table S5) for detecting a female-specific (W-linked) sequence (red asterisk) of the *Lj_Uch5l_W* fragment. Genomic DNAs of four males and four females of three *Leptidea* species were used as templates. A partial sequence of *Leptidea* ortholog of the *B. mori RpS5* gene was used as a positive control.

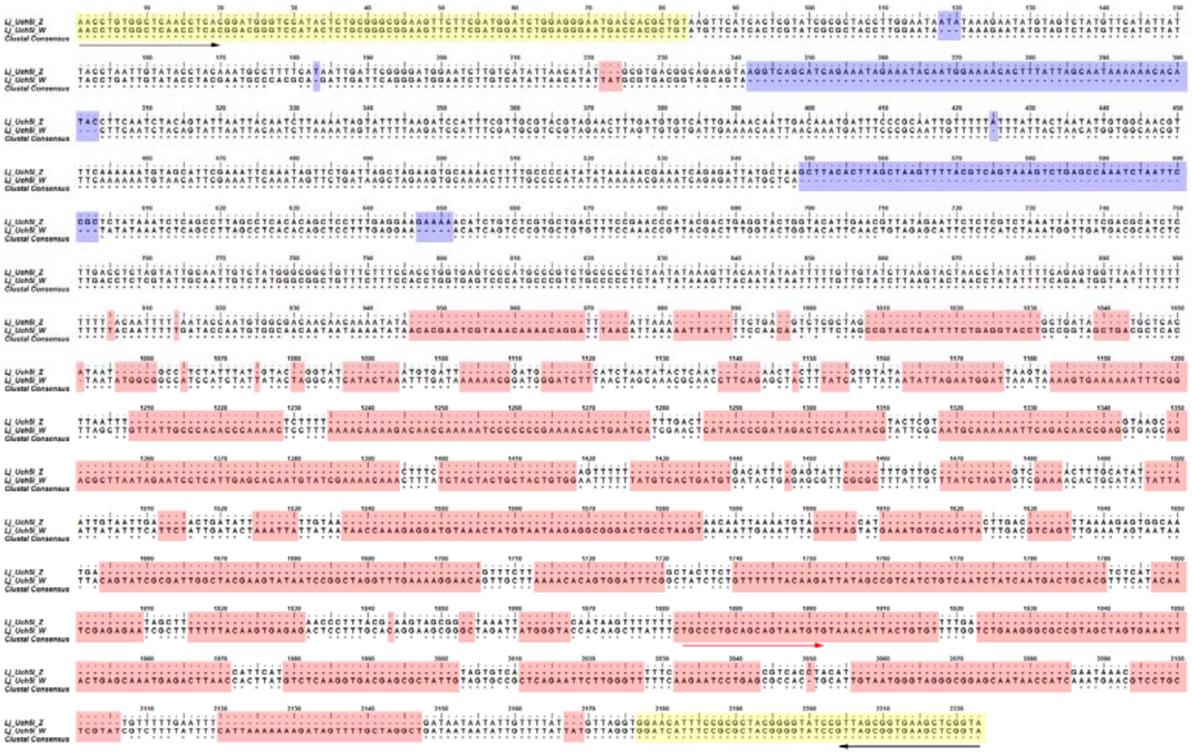

Fig. S7. Alignment of the female-specific (W-linked) partial sequence (*Lj_Uch5l_W*) and the common (Z_3-linked) partial sequence (*Lj_Uch5l_Z*) of the *Leptidea juvernica* ortholog of the *B. mori Uch5l* gene. Exon sequences are indicated in yellow. Black arrows indicate positions of primers for amplifying *Lj_Uch5l_Z* and *Lj_Uch5l_W*, red arrow a re-designed primer for detecting female-specific (W-linked) sequence. In the intron sequence, deletions in the W-linked sequence are indicated in blue, insertions in pink. Asterisks indicate Clustal consensus nucleotides.

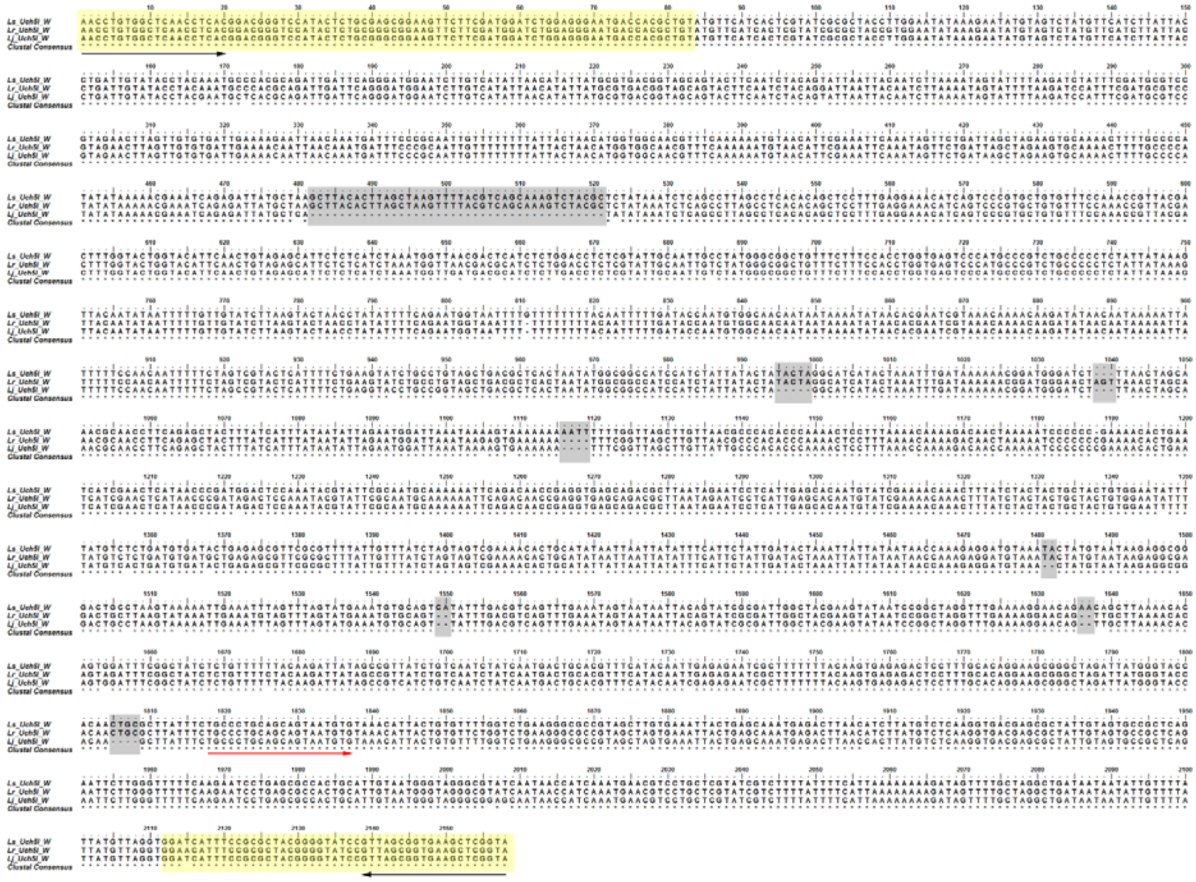

Fig. S8. Alignment of the female-specific (W-linked) partial sequences (*Ls_Uch5l_W*, *Lr_Uch5l_W*, and *Lj_Uch5l_W*) of the *Leptidea* ortholog of the *B. mori Uch5l* gene in *L. sinapis*, *L. reali*, and *L. juvernica*, respectively. Exon sequences are indicated in yellow. Black arrows indicate positions of primers for amplifying *Uch5l_W*, red arrow a re-designed primer for detecting female-specific (W-linked) sequence. Indels in the intron sequence are indicated in grey. Asterisks indicate Clustal consensus nucleotides.

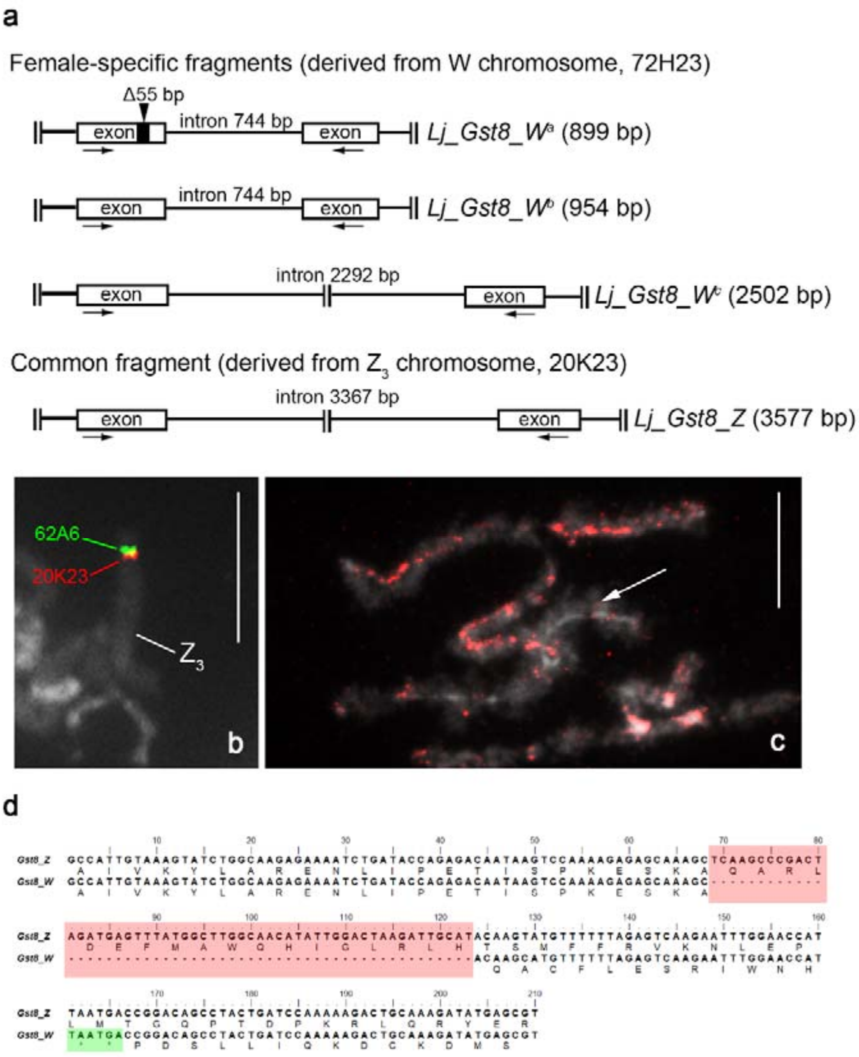

Fig. S9. Identification and characterization of a W-specific orthologous sequence of the *B. mori Gst8* gene in *Leptidea juvernica*. (**a**) Schematic illustrations of partial sequences of *Leptidea* orthologs of the *B. mori Gst8* gene. Three female-specific fragments (three upper schemes, *Lj_Gst8_W*$^{a\text{-}c}$) were obtained from genomic DNA of females and DNA of the 72H23 BAC clone, and common fragment (lower scheme) from genomic DNAs of both sexes and DNA of the 20K23 BAC clone. Δ55 bp indicates a deletion in the exon of the *Lj_Gst8_W*^{a} sequence, arrows the positions of designed primers. (**b**) BAC-FISH mapping of the 62A6 (green signals) and 20K23 (red signals) probes on male pachytene chromosomes of *L. juvernica*. Both BAC probes mapped to a single bivalent (Z_3 chromosome). (**c**) BAC-FISH mapping of the 72H23 probe (red signals) on the sex chromosome multivalent (arrow) in female pachytene of *L. juvernica*. (**b** and **c**) Chromosomes were stained with DAPI (grey). Bar = 10 µm. (**d**) Alignment of partial exon sequences and putative amino acids of the *Lj_Gst8* sequences derived from Z_3 and W chromosomes of *L. juvernica*. Pink and green boxes indicate W-specific deletion and putative stop codons in the *Lj_Gst8_W*^{a} sequence, respectively.

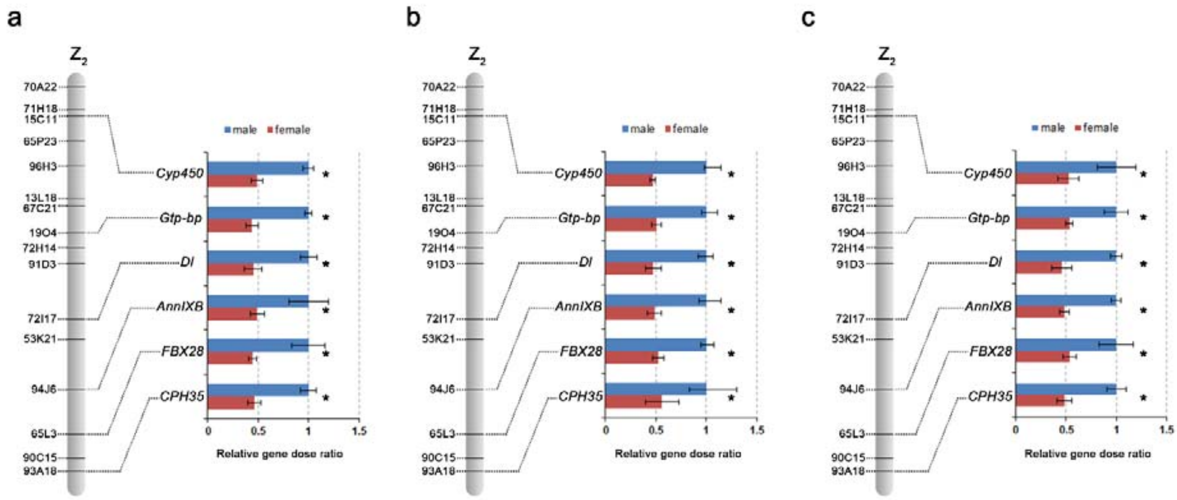

Fig. S10. Female-to-male gene dose ratios of Z-linked genes in three *Leptidea* species. Results of qPCR showing female (red columns) to male (blue columns) relative gene dose ratios of Z_2-linked genes normalized to the autosomal reference gene *RpS5* in *L. juvernica* (**a**), *L. sinapis* (**b**) and *L. reali* (**c**). Average of target to reference gene dose in male = 1. Error bars represent SDs calculated from three independent samples (Supplementary Tables S6, S7, S8). Asterisks indicate a significant twofold difference (female-to-male = 1:2) in unpaired two-tailed *t* test for unequal variances.

Table S1. List of *Leptidea juvernica* orthologs of selected *Bombyx mori* genes, designed primer sets for BAC screening, BAC clones used in this study, and summary of BAC-FISH mapping on *L. juvernica* chromosomes. *These primers were also used for qPCR in this study.

		Bombyx mori			*Leptidea juvernica* (Lj)						
Gene symbol	Gene description in KAIKObase	Gene ID in KAIKObase	Chrom. no.	Chromosome position in KAIKObase	Lj transcriptome assembly	Forward primer for BAC screening	Reverse primer for BAC screening	PCR product (bp)	BAC clone from Lj library	BAC-FISH mapping to chromosome	BAC- FISH result shown in
Tan	Tan	BMgn002077	Z	460237-480848	scaffold18132	ACGAAGGACTTGGTGTTGCC	AGAAGTAAGATCGGGTGCCAC	107	63E2	Z_1	Fig. 2
ap	apterous	BMgn002127	Z	3487639-3516414	C1003895	TGATAGACAATGGCACGCCG	CCGTCACGAGAGAAGCATGT	87	53C10	Z_1	Fig. 2
ABCF2	ATP-binding cassette sub-family F member 2	BMgn002004	Z	4621452-4632826	scaffold19631	AATCCTCTCTTTTGGCTGCG	TCGTCTTCGCAATGGGCAA	184	90D1	Z_1	Fig. 2
Prm	Paramyosin	BMgn000612	Z	5986799-6002013	C1077381	CTGAGGTTTACGAACTGTTGGC	TTGTGCGATGTTATGTCCACG	154	9J14	Z_1 + autosome	Fig. 2, Fig. S2a
ket	kettin	BMgn000622	Z	6513219-6533895	scaffold23153	AATGACGCGATTCGATTGCC	TGGTGTAGGTGTGCCAACTG	340	91P9	Z_1	Fig. 2
Idgf	Imaginal disk growth factor	BMgn000648	Z	8533563-8553629	C1067791	TGGATCCAGCACTGTCGTTC	GTGCGTCTTGTCTACGTCCA	113	93O2	Z_1	Fig. 2
Th	tyrosine hydroxylase	BMgn000563	Z	8795363-8803219	C1074465	CGTGCAGTTTGATGCTCTCG	CACTCGGAATGCAAGCGAAG	565	66E6	Z_1	Fig. 2
Tpi	Triosephosphate isomerase	BMgn000559	Z	9023502-9027095	scaffold6903	TCGAGTCTGGCCTGAAAGTT	CCCCACCAACTAAGAACCCG	1925<br>1526<br>1435	91C3<br>96D19<br>66L7	autosome<br>autosome<br>autosome	Fig. S2c
Ybp	Y box protein	BMgn000526	Z	10855404-10857772	C1061571	AGCGGAAAAAGTGTCTGGCA	ACCACTGCAAACTCAACCGC	330	66E6	Z_1	Fig. 2
Imp	IGF-II mRNA-binding protein	BMgn000515	Z	11419210-11500018	scaffold9755	GAGCACCATCCGCCTCATTA	CATTGGTGCAGTTTTCCGGG	120	69D15	Z_1	Fig. 2
per	period	BMgn000485	Z	12956618-13004501	scaffold14767	AAGACATTCCTCAACGGGGG	GGTACATGGCGATAGCGTCT	107	72D11	Z_1	Fig. 2
Pgd	6-phosphogluconate dehydrogenase	BMgn012298	Z	15112863-15127673	scaffold8411	CTTCAGAAGACGCACGGTTC	GGTTGGATGGAAGCATGGGA	130	62O17	autosome	Fig. S2b
Masc	Masculinizer (hypothetical protein KGM_08818 [*Danaus plexippus*])	BMgn012300	Z	15129206-15132406	scaffold19378	GACCCGCATGTAAATTCCGC	AACTGTCCCGCAACCTCAAA	160	62N7	Z_1	Fig. 2
SNF4Aγ	SNF4/AMP-activated protein kinase gamma subunit	BMgn012310	Z	15595590-15678086	scaffold8810	ACGATCATAGAGGCGTTGGG	CTTGAGCCGACCTTCAGCAT	81	90K6	Z_1	Fig. 2
Ldh	L-lactate dehydrogenase	BMgn012336	Z	17338625-17350610	scaffold20865	GTGGCCGGAGTAAGACTGAG	CTTGAGGCGGATCACTTCGT	120	95B19	Z_1	Fig. 2
Shkr	Shaker	BMgn003851	Z	20911282-20921258	C1074547	CTACCACCAATGGCACCAAG	TAGAAGCCTTGAGTGTGCGG	399	19P21	Z_1	Fig. 2
Hn	phenylalanine hydroxylase (henna)	BMgn003866	Z	21842454-21845665	scaffold10190	TGTCCCATGGTTTCCACGTC	TGGTGTAGACGACATGGGG	191	69F16	Z_1	Fig. 2
EH-dp1	EH domain-containing protein 1	BMgn009992	7	67746-96791	C1075193	TATCCATTGTGGACACGCCC	ACACCCTCATGAGTTGCTGG	252	72H14	Z_2	Fig. 3
KGM21114	hypothetical protein KGM_21114 [*Danaus plexippus*]	BMgn015300	7	1455398-1458517	C1073355	AATGCAGTGGTGGGTACTGG	GGTTTGTCCATCCTGCGAGA	227	91D3	Z_2	Fig. 3
Dl	Delta	BMgn010195	7	6075871-6111563	C1078355	GCACCTGTCATAACACCGGA*	CTTCATCGGGAACACAGGCT*	138	72I17	Z_2	Fig. 3
tRNAmt	tRNA methyltransferase	BMgn010207	7	7001096-7096151	C1062109	AGGCAGCGGTTCTCTTACAC	GAAATCTGCCAAGCCATGGC	133	53K21	Z_2	Fig. 3
AnnIXB	Annexin IX isoform B	BMgn010130	7	8615817-8626114	scaffold3030	GCGAACTCAAGAGCGAGCTA*	TCAGTACCAATTCCCGCGAC*	118	94J6	Z_2	Fig. 3
pixie	pixie	BMgn010129	7	8632389-8643051	scaffold12930	TAGATTGCCTATTCCGCGCC	TGGGTTTTTGTTTCCCAGCA	104	94J6	Z_2	Fig. 3
CUTClp	putative Copper homeostasis protein cutC-like protein	BMgn010253	7	8644140-8647781	C1055915	CCAGTCCGATTCCACTGACA	CCCATAGCGACAAATACCGC	244	94J6	Z_2	Fig. 3
Cad	Cadherin	BMgn010267	7	9747774-9767857	C1078555	TCTTTGGAGTTGGTCGAGGG	CTCATGCACGTGGACAGTTG	179	95N3	autosome	Fig. S3c
Gcy	Guanylate cyclase	BMgn010091	7	11414173-11492005	scaffold20527	TACGCACCCTGCGGTATCTA	CGACGCAAGCTCCGTAAAAC	154	41B20	autosome	Fig. S3c
unc50l	unc-50-like protein	BMgn008674	7	14646799-14653293	C1059873	GTAACGTTTGACCGCAGCAC	CCACCTCACCCACTCCTACA	101	42F24	autosome	Fig. S3c
Trp	Translocation protein Sec62	BMgn005270	8	4673007-4693568	scaffold11758	TCGTCTCGAGATGCACATGG	GATGGTTCCGAAGACGACGA	121	95D23	autosome	Fig. S3c
Eno	Enolase	BMgn005493	8	11726013-11734127	scaffold16989	CCAAGAAGGGTGTGCCACTA	ATAGCCAGCTTGTTCCCAGC	124	70D8	autosome	Fig. S3c
m5u-mt	RNA m5u methyltransferase	BMgn005286	8	13114526-13123939	scaffold16616	TACATATCGTGCAGTCCGGC*	GACGTGCTTCGTGTAAGGGA*	138	17J5	Z_3	Fig. 4
Gst8	Glutathione S-transferase 8	BMgn009935	8	15338482-15350404	scaffold10716	GCCATTGTAAAGTATCTGGC	ACGCTCATATCTTTGCAGTC	3577<br>899, 954, 2502	20K23<br>72H23	Z_3<br>W chromosomes	Fig. S9b<br>Fig. S9c
Uch5l	Ubiquitin carboxyl-terminal hydrolase 5-like isoform 1	BMgn009941	8	15351712-15371974	scaffold2960	AACCTGTGGCTCAACCTCAC	TACCGAGCTTCACCGCTAAC	1400<br>2095	62A6<br>1B2, 70F8	Z_3<br>W chromosomes	Fig. 4<br>Fig. 6a, b
KGM12964	hypothetical protein KGM_12964 [*Danaus plexippus*]	BMgn009934	8	15372779-15374302	C1045955	GTCTGATGGTTGCATGTTGCC	ATCAGTCCTGCTTTCTGTGCC	102	62A6	Z_3	Fig. 4
S3-12	putative plasma membrane associated protein, S3-12-like protein	BMgn009933	8	15377476-15398398	scaffold4813	AGGCGCGTTCCGAAGTATC*	GGCTTCGTTGGCAGTCTTCT*	172	62A6	Z_3	Fig. 4
Frl	Formin-like protein CG32138-like isoform 1	BMgn009881	8	16772354-16799660	scaffold25478	TGTTCGGCTTCAGAGGGAAC*	GCGAAAACTCGACACACCAG*	120	70B3	Z_4	Fig. 4
Lgr	Leucine-rich repeat G protein-coupled receptor precursor	BMgn009886	8	17100494-17156828	C1075353	CTTTGGAGCGATGGTTTGCC*	CACCGGCAGACAAATTGACG*	170	69P11, 90N21	Z_4	Fig. 4
KGM00143	hypothetical protein KGM_00143 [*Danaus plexippus*]	BMgn009852	8	17158743-17162365	C1059283	CATGGAGGCAAAACCCGTAG	CAGTCTCAGCTTCGTTGGTC	205	69P11, 90N21	Z_4	Fig. 4
tra2	transformer 2	BMgn009888	8	17184249-17192522	scaffold13829	CTAAAAACGAGTGCACCGGC	GCCCATGTATATGCCGGGAG	104	90N21	Z_4	Fig. 4
Ann1	Annexin isoform 1	BMgn009900	8	17604017-17616920	scaffold18408	AGCCAACTGTTGTGGGTGTT*	GACCGTGTAGTCAGGATCTCAA*	116	67E14	Z_4	Fig. 4
Dbadrh	putative DEAD box ATP-dependent RNA helicase	BMgn009910	8	18103635-18113027	C1072777	CGTCATGTACCATGCGCCTT*	GCCTCTGTATTGGTGTGGGT*	121	65A14	Z_4	Fig. 4
Smc	Structural maintenance of chromosomes 1A	BMgn009835	8	18335387-18355471	scaffold10022	TTGAGCGGCTTGGCTTGTAT*	CGACACACAAGCGGTACAAC*	172	22K17	Z_4	Fig. 4
KGM08377	hypothetical protein KGM_08377 [*Danaus plexippus*]	BMgn001710	11	76987-81275	C1073233	GAAGGGTCACGGGGTTAAGG	GGGAACAGGCTATTGGTGCT	162	70A22	Z_2	Fig. 3
Zf228	putative zinc finger protein 228	BMgn001744	11	891908-909137	C1075469	CACTTCAAACACTCGCACGC	TGGTTCCTTTGTGCCACCTC	300	71H18	Z_2	Fig. 3
Cyp450	Cytochrome P450	BMgn001753	11	1264947-1272550	C1074719	TTCTGCGGGTCTTACGTGAC*	CAACCTCTGCATCTCTGGCA*	141	15C11	Z_2	Fig. 3
Pisd	Phosphatidylserine decarboxylase	BMgn001766	11	1795166-1796032	scaffold26082	TGGGGATTGGGAGTATGGGT	AGTTGGAAGTTAGCAGGGGC	231	65P23	Z_2	Fig. 3
KGM01846	hypothetical protein KGM_01846 [*Danaus plexippus*]	BMgn001655	11	2284069-2284887	C1059407	CAAGCCTTCCACAGAGGCAT	GTCTTCCAGGAATGCTGGCT	371	96H3	Z_2	Fig. 3
Cpsf5	Cleavage and polyadenylation specific factor 5	BMgn001806	11	3052181-3055687	scaffold555	ACCCCCGCAATACCCATACA	GAGGCGCAGCCACTAACTTAT	131	67C21	Z_2	Fig. 3
Osbp	Oxysterol binding protein	BMgn001810	11	3139662-3192524	scaffold25684	GAGCATACCCTGGCCATTGA	ACCAGTTCAGGAGGGTCTGT	169	13L18	Z_2	Fig. 3
Gtp-bp	putative GTP-binding protein	BMgn011993	11	5217859-5228534	C1074167	ATGTTCGGAGCTGATGACGG*	CACAAGCATGGCGTAGTGTG*	145	19O4	Z_2	Fig. 3
RpL18	Ribosomal protein L18	BMgn011620	11	9399516-9401192	C1046021	GGCACGTCACATGAAGAAGC	AGCAGCTAAGATACGGGCTC	151	95K24	autosome	Fig. S3b
Dmc1	Dmc1 homolog	BMgn011811	11	11626450-11635244	C1060269	CTGACCCCAAGAAGCCCATC	GCTATTCTGTTCTCGCCCCG	94	43I3	autosome	Fig. S3b
KGM19656	hypothetical protein KGM_19656 [*Danaus plexippus*]	BMgn012129	11	16517488-16577040	scaffold24981	GTGTTCTCGACGCACTCGGT	CATCTTGTCCACGCGCTTCT	105	43L15	autosome	Fig. S3b
RpS5	Ribosomal protein S5	BMgn007710	15	7586843-7587692	C1050213	TTGGTCGTGCTGGTACTGTC*	TCTGAAGGCTGCTTCACGAG*	110	9M16	autosome	Fig. S3c
RpS8	Ribosomal protein S8	BMgn003397	15	13998108-14000483	C1043175	GCATCCAACAATGAGTTAGTCCG	TGCCGAGTGGCAACAAGTAG	115	96L18	autosome	Fig. S3c
RpP1	Ribosomal protein P1	BMgn003412	15	14486757-14488166	C1032998	ACCCTACTGGCCTGGCTTAT	CACTCCTGATCCGATGTTTGTG	82	89C2	autosome	Fig. S3c
Ctatpase	putative cation-transporting atpase	BMgn003317	15	15694441-15712206	C1077515	ATCATGTCAGCAGGGACACG*	ACACGCCCTCTGATGGTTAC*	115	41J22	Z_3	Fig. 4
RpP0	Ribosomal protein P0	BMgn003309	15	16146287-16150050	C1063525	AATGTTTCATCGTGGGTGCC*	CTCAACAAGGTCTCCACGGG*	215	17I6	Z_3	Fig. 4
Top2-bp1	DNA topoisomerase 2 binding protein 1	BMgn003443	15	16724374-16748667	scaffold2451	ACAACATTCGGGAGGTGGTA*	GACATTTCGTTGCCCTCAGT*	143	19O6	Z_3	Fig. 4
Treh	Trehalase	BMgn005664	17	1555037-1558124	scaffold6145	ACCTTCCACCGTCGTGTATC	GGTAATACCAGCGACTGCCA	585	66E20	autosome	Fig. S3a
Rrb	putative regulator of ribosome biosynthesis	BMgn005564	17	2388316-2390458	scaffold14373	CCTTCTCTGCTTGTGGGGTT	CTTCAACCGCATCATTGGGC	138	65E15	autosome	Fig. S3a
eIF3D	eukaryotic translation initiation factor 3 subunit D	BMgn005592	17	4290839-4299895	scaffold25814	TAGCTCGGTGTGAGCATGAC	TCATTGGCTAGCACAGCTCC	157	93F8	autosome	Fig. S3a
ASPG	l-asparaginase	BMgn007025	17	11551636-11556300	scaffold6287	CTTGACGTTGGTGCTGTGAC	GCCACCCAACAAGGAATGTG	99	92J7	Z_1	Fig. 2
RpL22	Ribosomal protein L22	BMgn006986	17	14152955-14155752	C1035615	TATTGACTGCACACACCCCG	GTCACGAGCGATAACAACGTG	138	19D3	Z_1	Fig. 2
KGM04993	hypothetical protein KGM_04993 [*Danaus plexippus*]	BMgn003962	17	18278392-18281478	C1053921	AAAGGCGAACGAATGATGGC	GTCCGCGAGTCAGTTTAGGT	100	94M23	Z_1	Fig. 2
FBX28	putative F-box protein 28	BMgn008185	24	1656904-1669541	scaffold15014	GTGGAAACCTCCAAAACGCC*	GTTCTTGCGGAATGCTGGTG*	117	65L3	Z_2	Fig. 3
CPH35	putative cuticle protein CPH35	BMgn000083	24	7542521-7548473	C1078471	AACTCCAGCGAACCTCCAAG*	ACTCGGTATCAGAGGTGGCT*	174	93A18	Z_2	Fig. 3
Tmc7	Transmembrane channel-like protein 7	BMgn000078	24	8061419-8067032	C1077097	GGCTTTGCTGTCACTTGTAGC	TACAAAAGTCCCAACCGCCG	125	90C15	Z_2	Fig. 3
KGM02279	hypothetical protein KGM_02279 [*Danaus plexippus*]	BMgn009578	24	11525730-11537948	scaffold12197	GGTGCCATAGGACGAAAGGT	TTCCCAGGTTTGGTTCTGCC	209	49I8	autosome	Fig. S3b
Sui1	Protein translation factor SUI1 homolog	BMgn003805	24	16081319-16082543	C1057163	GGAACGGGCGCAAAACTCTA	CCACTGGCAAATGTTCTCGC	176	94G12	autosome	Fig. S3b
O-fut2	Protein-O-fucosyltransferase 2	BMgn012194	24	17459857-17461971	C1073039	GCTCTGGGATGTGATTGCCT	TCCTGTCGTCTCCAATGCAC	228	49E5	autosome	Fig. S3b

Table S2. Z chromosome scaffolds in the assembled genome sequence of *Leptidea sinapis*. Yellow, purple, green, and red represent Z_1-, Z_2-, Z_3-, and Z_4-linked BAC clones or scaffolds in the genome sequence, respectively. *A indicates autosome.

Bombyx mori			***Leptidea juvernica***		***Leptidea sinapis***	
**Gene symbol**	**Chr. No.**	**Chromosome position in KAIKObase**	**BAC clone**	**Chr. No.**	**Scaffold of genome sequence (Accession No. PRJEB21838)**	**Chr. No.**
Ldh	Z	17338625-17350610	95B19	Z_1	scaffold_176	Z_1
SNF4Aγ	Z	15595590-15678086	90K6	Z_1	scaffold_176	Z_1
Masc	Z	15129206-15132406	62N7	Z_1	scaffold_565	Z_1
Hn	Z	21842454-21845665	69F16	Z_1	scaffold_26	Z_1
Shkr	Z	20911282-20921258	19P21	Z_1	scaffold_117	Z_1
per	Z	12956618-13004501	72D11	Z_1	scaffold_0, contig: 11201	Z_1
Tan	Z	460237-480848	63E2	Z_1	scaffold_0, contig: 11121	Z_1
ap	Z	3487639-3516414	53C10	Z_1	scaffold_0, contig: 11121	Z_1
ket	Z	6513219-6533895	91P9	Z_1	scaffold_330	Z_1
Prm	Z	5986799-6002013	9J14	Z_1 + A*	scaffold_273, contig: 9531	Z_1 + A*
ABCF2	Z	4621452-4632826	90D1	Z_1	scaffold_49	Z_1
Imp	Z	11419210-11500018	69D15	Z_1	scaffold_86	Z_1
Idgf	Z	8533563-8553629	93O2	Z_1	scaffold_542	Z_1
Th	Z	8795363-8803219	66E6	Z_1	scaffold_55	Z_1
Ybp	Z	10855404-10857772	66E6	Z_1	scaffold_55	Z_1
ASPG	17	11551636-11556300	92J07	Z_1	scaffold_14	Z_1
KGM04993	17	18278392-18281478	94M23	Z_1	scaffold_162	Z_1
RpL22	17	14152955-14155752	19D03	Z_1	scaffold_944	Z_1
KGM08377	11	76987-81275	70A22	Z_2	scaffold_629	Z_2
Zf228	11	891908-909137	71H18	Z_2	scaffold_682	Z_2
Cyp450	11	1264947-1272550	15C11	Z_2	scaffold_474	Z_2
Pisd	11	1795166-1796032	65P23	Z_2	scaffold_407	Z_2
KGM01846	11	2284069-2284887	96H3	Z_2	scaffold_936	Z_2
Osbp	11	3139662-3192524	13L18	Z_2	scaffold_178	Z_2
Cpsf5	11	3052181-3055687	67C21	Z_2	scaffold_516	Z_2
Gtp-bp	11	5217859-5228534	19O4	Z_2	scaffold_113	Z_2
EH-dp1	7	67746-96791	72H14	Z_2	scaffold_260	Z_2
KGM21114	7	1455398-1458517	91D3	Z_2	scaffold_48	Z_2
Dl	7	6075871-6111563	72I17	Z_2	scaffold_3	Z_2
tRNAmt	7	7001096-7096151	53K21	Z_2	scaffold_3	Z_2
AnnIXB	7	8615817-8626114	94J6	Z_2	scaffold_73	Z_2
pixie	7	8632389-8643051	94J6	Z_2	scaffold_73	Z_2
CUTClp	7	8644140-8647781	94J6	Z_2	scaffold_73	Z_2
FBX28	24	1656904-1669541	65L3	Z_2	scaffold_335	Z_2
Tmc7	24	8061419-8067032	90C15	Z_2	scaffold_317	Z_2
CPH35	24	7542521-7548473	93A18	Z_2	scaffold_317	Z_2
Top2-bp1	15	16724374-16748667	19O6	Z_3	scaffold_648	Z_3
RpP0	15	16146287-16150050	17I6	Z_3	scaffold_329	Z_3
Ctatpase	15	15694441-15712206	41J22	Z_3	scaffold_39	Z_3
m5u-mt	8	13114526-13123939	17J5	Z_3	scaffold_39	Z_3
Uch5l	8	15351712-15371974	62A6	Z_3	scaffold_18	Z_3
KGM12964	8	15372779-15374302	62A6	Z_3	scaffold_18	Z_3
S3-12	8	15377476-15398398	62A6	Z_3	scaffold_18	Z_3
Lgr	8	17100494-17156828	69P11, 90N21	Z_4	scaffold_18	Z_3
KGM00143	8	17158743-17162365	69P11, 90N21	Z_4	scaffold_18	Z_3
tra2	8	17184249-17192522	90N21	Z_4	scaffold_18	Z_3
Frl	8	16772354-16799660	70B3	Z_4	scaffold_567, scaffold_1238	Z_3
Ann1	8	17604017-17616920	67E14	Z_4	scaffold_567	Z_3
Dbadrh	8	18103635-18113027	65A14	Z_4	scaffold_193	Z_3
Smc	8	18335387-18355471	22K17	Z_4	scaffold_193	Z_3

Table S3. List of sequences in sub-clones derived from W-BAC clones.

Sub-clone No.	Insert size (bp)	BAC clone	Accession No.	Accession No. of NCBI best hit	NCBI best hit species	NCBI best hit gene description	NCBI e-value	NCBI identities
2	748	1B2	LC510273	XM_013317671.1	*Papilio xuthus*	RNA-directed DNA polymerase from mobile element jockey-like (LOC106121843), partial mRNA	2e-52	210/256 (82%)
3	853	1B2	LC510274	XP_013177582.1	*Papilio xuthus*	uncharacterized protein LOC106125042	6e-17	35/80 (44%)
9	1832	1B2	LC510275	XP_013188213.1	*Amyelois transitella*	uncharacterized protein LOC106133134	0.0	284/410 (69%)
11	1691	1B2	LC510276	XP_022128388.1	*Pieris rapae*	ubiquitin carboxyl-terminal hydrolase 5	3e-23	48/59 (81%)
14	1501	1B2	LC510277	XP_013197438.1	*Amyelois transitella*	uncharacterized protein LOC106140399, partial	3e-07	30/46 (65%)
18	2370	1B2	LC510278	XP_008486323.1	*Diaphorina citri*	ubiquitin carboxyl-terminal hydrolase 5-like, partial	2e-25	49/59 (83%)
35	977	1B2	LC510279	FP565803.1	*Heliconius numata*	DNA sequence from clone AEHN-7C9, complete sequence	4e-151	224/293 (76%)
80	1013	1J4	LC510280	XR_960789.1	*Plutella xylostella*	uncharacterized LOC105387889, ncRNA	1e-62	114/243 (47%)

Table S4. GenBank accession numbers of common (Z_3-linked) and female-specific (W-linked) sequences of *Leptidea* orthologs of the *B. mori Uch5l* and *Gst8* genes used in this study.

***Leptidea* Species**	**Symbol**	**Chromosome**	**Accession No.**	**Fragment size (bp)**
L. juvernica	*Lj_Uch5l_Z*	Z_3	LC510285	1400
L. juvernica	*Lj_Uch5l_W*	W	LC510286	2095
L. sinapis	*Ls_Uch5l_W*	W	LC510287	2154
L. reali	*Lr_Uch5l_W*	W	LC510288	2149
L. juvernica	*Lj_Gst8_Z*	Z_3	LC510281	3577
L. juvernica	*Lj_Gst8_W*[a]	W	LC510284	899
L. juvernica	*Lj_Gst8_W*[b]	W	LC510283	954
L. juvernica	*Lj_Gst8_W*[c]	W	LC510282	2502

Table S5. List of additional primer sets used for the identification of W chromosome and qPCR.

Gene symbol	Forward primer	Reverse primer	Result shown in
Uch5l_W	TGCCCTGCAGCAGTAATGTG	TACCGAGCTTCACCGCTAAC	Supplementary Fig. S6c
Gst8	TGACCGGACAGCCTACTGAT	GTCACCGGCAACAAAGTCAC	Fig. 7 (main text; for qPCR)
Uch5l	GTTAGCGGTGAAGCTCGGTA	GCTGCACGTTGATTCCGAAG	Fig. 7 (main text; for qPCR)

Table S6. Result of quantitative PCR in *Leptidea juvernica*. Female to male relative dose ratios of target genes were determined in three different specimens (Sample 1-3) by comparison with the autosomal reference gene *Ribosomal protein S5* (*RpS5*). PCR efficiencies (E) were calculated from the slope of standard curve. PCR efficiencies ($E_{reference}$) in males and females were 1.0666 and 1.0286, respectively. Null hypothesis (H_0) of no difference (female to male = 1:1) or a twofold difference (female to male = 1:2) was tested by unpaired two-tailed t test for unequal variances.

Target Gene Symbol	Sex	Target to reference gene does ratio				*P* value of *t* test	
		Sample 1	Sample 2	Sample 3	E_{target}	H_0 (1:1)	H_0 (1:2)
Cyp450	Male	0.942896	1.025323	1.034369	1.062	<0.001	0.8090
	Female	0.450407	0.550651	0.468694	1.073		
Gtp-bp	Male	1.041483	0.981610	0.978157	1.003	<0.001	0.2431
	Female	0.402733	0.511980	0.407777	1.039		
Dl	Male	1.000931	0.922020	1.083566	1.040	0.0013	0.5466
	Female	0.393518	0.555461	0.422895	1.071		
AnnIXB	Male	0.992102	0.826371	1.219744	1.111	0.0233	0.8838
	Female	0.493092	0.564189	0.423176	1.050		
FBX28	Male	0.965259	0.868079	1.193429	1.075	0.0297	0.6002
	Female	0.459318	0.479180	0.405234	1.069		
CPH35	Male	1.051717	0.910989	1.043730	1.043	<0.001	0.5307
	Female	0.405691	0.532765	0.446580	1.147		
Top2-bp	Male	1.029147	0.905910	1.072599	1.015	0.0739	
	Female	0.793182	0.931669	0.762507	1.048		
RpP0	Male	1.044840	0.966642	0.990113	1.015	0.3150	
	Female	0.682671	1.052519	0.827250	1.101		
Ctatpase	Male	1.069421	0.961730	0.972295	1.115	<0.001	0.1126
	Female	0.406666	0.450494	0.372143	1.065		
m5u-mt	Male	0.977715	0.899824	1.136659	1.024	0.0058	0.6244
	Female	0.445529	0.525223	0.418286	0.978		
Gst8	Male	1.039991	0.928505	1.035586	1.064	0.0296	0.0187
	Female	2.286304	3.408173	2.864177	1.045		
Uch5l	Male	0.961634	0.960260	1.082932	1.054	0.0036	0.0030
	Female	5.781697	6.199966	5.214364	1.056		
S3-12	Male	1.072958	0.903912	1.031077	0.990	0.6571	
	Female	0.743974	1.153428	0.920497	1.007		
Lgr	Male	1.006657	0.961038	1.033660	1.105	0.0019	0.6169
	Female	0.391156	0.552301	0.472599	1.169		
Frl	Male	1.062552	0.977307	0.962983	1.131	0.1097	
	Female	0.858387	0.957948	0.779893	1.129		
Ann1	Male	1.043234	0.955702	1.002988	0.996	0.0025	0.5799
	Female	0.367927	0.535616	0.493721	1.001		
Dbadrh	Male	1.003272	0.904545	1.101923	0.984	0.0024	0.1979
	Female	0.419860	0.432216	0.346205	1.016		
Smc	Male	1.036803	0.806655	1.195682	1.089	0.9851	
	Female	0.823278	1.272034	0.933302	1.002		

Table S7. Result of quantitative PCR in *Leptidea sinapis*. Female to male relative dose ratios of target genes were determined in three different specimens (Sample 1-3) by comparison with the autosomal reference gene *Ribosomal protein S5* (*RpS5*). PCR efficiencies (E) were calculated from the slope of standard curve. PCR efficiencies ($E_{reference}$) in males and females were 1.0119 and 1.0292, respectively. Null hypothesis (H_0) of no difference (female to male = 1:1) or a twofold difference (female to male = 1:2) was tested by unpaired two-tailed *t* test for unequal variances.

Target Gene Symbol	Sex	Target to reference gene does ratio				*P* value of *t* test	
		Sample 1	Sample 2	Sample 3	E_{target}	H_0 (1:1)	H_0 (1:2)
Cyp450	Male	0.963946	0.886818	1.169804	0.9695	0.0242	0.6891
	Female	0.484107	0.477752	0.439950	0.9682		
Gtp-bp	Male	1.099556	0.885733	1.026786	1.0085	0.0053	0.9560
	Female	0.476108	0.560890	0.487301	0.9432		
Dl	Male	1.047208	0.929199	1.027681	1.0086	<0.001	0.6970
	Female	0.393724	0.552443	0.484199	1.0558		
AnnIXB	Male	1.077823	0.842065	1.101810	1.0833	0.0113	0.8527
	Female	0.426951	0.568507	0.469935	1.0988		
FBX28	Male	1.079850	0.925612	1.000478	1.0480	<0.001	0.6976
	Female	0.461579	0.565944	0.547212	0.9936		
CPH35	Male	1.247026	1.167234	0.843739	1.0744	0.0288	0.8791
	Female	0.610955	0.611803	0.469748	1.0436		
Top2-bp	Male	1.105132	0.932362	0.970512	1.0645	0.0054	<0.001
	Female	1.339468	1.561362	1.524792	0.9743		
RpP0	Male	1.011694	0.857674	1.152467	1.0430	0.7351	
	Female	0.984024	1.066503	0.857502	1.0164		
Ctatpase	Male	1.052916	0.819506	1.158922	1.0904	0.0163	0.6857
	Female	0.384830	0.556926	0.439169	1.0768		
m5u-mt	Male	1.094686	0.906662	1.007546	0.9856	0.0047	0.4753
	Female	0.454951	0.633134	0.603542	0.9042		
Gst8	Male	1.046260	0.937662	1.019329	0.9780	0.0110	0.0073
	Female	3.622486	2.841745	3.104228	0.9845		
Uch5l	Male	0.995279	0.874452	1.148997	0.9680	0.0079	0.0071
	Female	9.726574	12.009422	9.333969	0.9447		
S3-12	Male	1.076652	0.931444	0.997168	0.9223	0.6388	
	Female	0.886047	1.040777	0.984586	0.8659		
Lgr	Male	0.938506	0.818977	1.301042	1.0549	0.9565	
	Female	0.964994	1.179988	0.942533	0.9817		
Frl	Male	1.044224	0.909999	1.052363	0.9879	0.9416	
	Female	0.937354	1.143279	0.945053	0.9901		
Ann1	Male	1.018312	0.930790	1.055036	0.9610	<0.001	0.7346
	Female	0.439031	0.552394	0.457180	0.9624		
Dbadrh	Male	1.070411	0.927587	1.007151	0.9969	0.3950	
	Female	0.915753	1.001026	0.944868	0.9620		
Smc	Male	0.975944	0.878512	1.166346	0.9588	0.6373	
	Female	0.985399	0.987854	0.900114	0.9510		

Table S8. Result of quantitative PCR in *Leptidea reali*. Female to male relative dose ratios of target genes were determined in three different specimens (Sample 1-3) by comparison with the autosomal reference gene *Ribosomal protein S5* (*RpS5*). PCR efficiencies (E) were calculated from the slope of standard curve. PCR efficiencies ($E_{reference}$) in males and females were 1.0839 and 1.1194, respectively. Null hypothesis (H_0) of no difference (female to male = 1:1) or a twofold difference (female to male = 1:2) was tested by unpaired two-tailed t test for unequal variances.

Target Gene Symbol	Sex	Target to reference gene does ratio				*P* value of *t* test	
		Sample 1	Sample 2	Sample 3	E_{target}	H_0 (1:1)	H_0 (1:2)
Cyp450	Male	0.856418	0.955024	1.222641	1.0140	0.0304	0.8872
	Female	0.647794	0.474648	0.469080	1.0716		
Gtp-bp	Male	0.886748	1.008050	1.118709	1.0161	0.0214	0.7245
	Female	0.495384	0.568728	0.534816	1.0549		
Dl	Male	1.025807	0.937347	1.039999	1.0654	0.0039	0.5692
	Female	0.502879	0.530620	0.341028	1.0327		
AnnIXB	Male	0.950556	1.009898	1.041704	1.0654	<0.001	0.6932
	Female	0.535815	0.479994	0.436383	1.1164		
FBX28	Male	0.950756	0.875805	1.200945	0.9950	0.0208	0.8105
	Female	0.593318	0.554228	0.462843	1.0246		
CPH35	Male	0.992798	0.911647	1.104871	1.0290	0.0017	0.7811
	Female	0.554774	0.480578	0.411942	1.0421		
Top2-bp	Male	0.927544	0.859877	1.253801	1.0549	0.0242	0.0044
	Female	1.636343	1.711601	1.473737	1.0558		
RpP0	Male	0.910869	0.925785	1.185859	1.0561	0.5083	
	Female	1.151628	1.115623	0.985579	1.0794		
Ctatpase	Male	1.050023	1.133752	1.040799	1.1453	0.0038	0.5052
	Female	0.597772	0.593388	0.605332	1.2713		
m5u-mt	Male	0.886825	0.954338	1.181569	1.0226	0.0267	0.5830
	Female	0.682229	0.556403	0.480610	1.0126		
Gst8	Male	1.027546	0.919375	1.058536	1.0445	0.0060	0.0045
	Female	3.732209	4.574318	4.223017	1.0377		
Uch5l	Male	0.901601	0.907003	1.222859	1.0412	0.0051	0.0044
	Female	8.786450	8.452587	7.146103	1.0149		
S3-12	Male	0.884218	1.048882	1.078234	1.0401	0.0080	0.2713
	Female	0.607221	0.625690	0.542418	1.0209		
Lgr	Male	1.070322	0.960451	0.972768	1.0509	0.0036	0.0032
	Female	9.192332	11.099909	10.030911	1.1307		
Frl	Male	1.028702	0.931984	1.043042	1.0704	0.8630	
	Female	1.090150	0.960617	0.918593	1.1473		
Ann1	Male	0.964299	0.988965	1.048592	1.0466	0.0028	0.6721
	Female	0.593928	0.543655	0.437733	1.0610		
Dbadrh	Male	1.193716	0.798323	1.049349	0.9673	0.9150	
	Female	1.028459	1.190776	0.872422	1.0065		
Smc	Male	0.897704	0.893555	1.246652	0.9732	0.9186	
	Female	1.370668	0.623896	0.961619	1.0802		
